# Supplementary material for: Lavandula stoechas subsp. luisieri (Rozeira) Rozeira: Variability of Chemical Composition of Essential Oil in Wild Populations
Source: Plants (Basel). 2025 Nov 10;14(22):3435. doi: 10.3390/plants14223435 (PMC12656282; doi:10.3390/plants14223435)
Supplement: Supplementary file 1 [file plants-14-03435-s001.zip › Suplement S1. Checklist samples.pdf]

plants

MDPI

Table S1. Checklist of collected populations of *L. stoechas* subsp. *luisieri*. (Note. IDs: identification code of each population; C.: Country; Elev.: Elevation over sea level in meters; IDz: identification code of each zone).

IDs	Date	C.	Location	UTM	Elev.	Voucher	IDz	Zone
1	2024-04-26	ES	Alconera. Sierra Gorda	29SQC1854	608	HSS087666	1	Alconera
2	2024-05-14	PT	Aljezur. Monte Novo, Riberia da Cerca	29SNB2129	71	HSS087910	2	Atlantic coast
3	2024-05-15	PT	Aljezur. Bordeira. Barranco do Vale da Água	29SNB1014	47	HSS087881		
4	2024-05-15	PT	Aljezur. Barranco do Monte o Clérigo	29SNB1629	109	HSS087882		
5	2024-05-15	PT	Aljezur. Rogil. Praia da Barradinha	29SNB1638	38	HSS087883		
6	2024-05-15	PT	Odemira. São Salvador e Santa Maria. Alto de Bonesinho	29SNB3060	112	HSS087886		
7	2024-05-15	PT	Odemira. São Luís. Ribeira do Torgal	29SNB3265	36	HSS087903		
8	2024-05-15	PT	Odemira. Colos. Barranco da Formiga	29SNB3984	100	HSS087875		
9	2024-05-09	PT	Reguengos de Monsaraz. Alqueva	29SPC2741	192	HSS087898	3	Guadiana International
10	2024-05-14	PT	Mértola. Santana de Cambas	29SPB3074	194	HSS087913		
11	2024-05-21	ES	Garbayuela. Puerto de los Carneros	30SUJ2228	543	HSS088244	4	Montes de Toledo
12	2024-05-21	ES	Garbayuela. El Collado	30SUJ2930	525	HSS088246		
13	2024-05-21	ES	Fuenlabrada de los Montes. Morra del Verdugo	30SUJ4036	658	HSS088247		
14	2024-05-21	ES	Villarta de los Montes. Morro de la Hoya del Pozo	30SUJ4948	725	HSS088248		
15	2024-05-21	ES	Helechosa de los Montes. Morro Coral de Canto	30SUJ4551	517	HSS088249		
16	2024-05-21	ES	Helechosa de los Montes. Pipaón	30SUJ3056	515	HSS088250		
17	2024-05-21	ES	Helechosa de los Montes. Laguna	30SUJ5159	610	HSS088259		
18	2024-05-21	ES	Navalpino. Cerro de la Moheda	30SUJ6244	768	HSS088260		
19	2024-05-21	ES	Puebla de Don Rodrigo. Collado de los Pilones	30SUJ6633	824	HSS088264		
20	2024-05-21	ES	Saceruela. Alcudia	30SUJ5821	623	HSS088266		
21	2024-05-28	PT	Vila Velha de Ródão. Fratel	29SPD0279	204	HSS088433	5	Rio Tejo
22	2024-05-28	PT	Vila Velha de Ródão. Portela da Milhariça	29SPD1094	373	HSS088429		
23	2024-05-28	PT	Castelo Branco. Sobral do Campo. Nogueirão	29SPN2328	479	HSS088412	6	Serra da Gardunha
24	2024-05-28	PT	Castelo Branco. São Vicente da Beira. Mastro	29TPE2124	609	HSS088430		
25	2024-05-28	PT	Fundão. Lavacolhos. Quinta da Filhadeira	29TPE1643	469	HSS088424		
26	2024-05-28	PT	Fundão. Barroca. Vale de Alvaro	29TPE1242	617	HSS088417		
27	2024-05-28	PT	Fundão. Bogas de Cima. Bogas de Meio	29TPE0737	425	HSS088416		
28	2024-05-28	PT	Oleiros. Orvalho. Penedo das Sardas	29TPE0231	493	HSS088404	7	Serra de Alvelos
29	2024-05-28	PT	Oleiros. Cambas. Lomba da Corga Cega	29SNE9828	311	HSS088408		
30	2024-05-28	PT	Oleiros. Milrico	29SNE9622	547	HSS088398		
31	2024-05-28	PT	Oleiros. Isna. Pico Fornos	29SNE9810	829	HSS088402		
32	2024-05-28	PT	Proença-a-Nova. Zambuiero	29SNE9501	406	HSS088400		
33	2024-05-14	PT	Silves. São Bartolomeu de Messines. Campilhos	29SNB6424	144	HSS087895	8	Serra de Monchique
34	2024-05-14	PT	Silves. São Bartolomeu de Messines. Vales	29SNB6528	168	HSS087896		
35	2024-05-14	PT	Silves. São Marcos da Serra, Cerro do Moinho	29SNB5434	152	HSS087897		
36	2024-05-14	PT	Monchique. Alferce. Foz de Açor	29SNB4933	104	HSS087876		
37	2024-05-14	PT	Monchique. Relva Grande	24SNB4230	668	HSS087877		

Table S1. (Continued).

IDs	Date	C.	Location	UTM	Elev.	Voucher	IDz	Zone
38	2024-05-14	PT	Monchique. Fóia	24SNB3529	764	HSS087908	8	Serra de Monchique
39	2024-05-14	PT	Monchique. Fontainhas	24SNB3426	354	HSS087909		
40	2024-04-25	PT	Portalegre. Alegrete	29SPD4547	526	HSS087663	9	Serra de São Mamede
41	2024-04-25	ES	La Codosera	29SPD5346	362	HSS087665		
42	2024-04-30	ES	Aliseda. Mina Pastora	29SPD9865	362	HSS087674	10	Sierra de San Pedro
43	2024-04-30	ES	Cáceres. Puerto Durana	29SPD9455	365	HSS087673		
44	2024-04-30	ES	Herreruela. Morrón de Calabazones	29SPD8162	496	HSS087671		
45	2024-04-30	ES	Salorino. Puerto de Elice	29SPD6867	455	HSS087670		
46	2024-04-30	ES	San Vicente de Alcántara. Puerto de Elice	29SPD6966	500	HSS087669		
47	2024-05-09	ES	Higuera de la Sierra	29SQB2488	468	HSS087915	11	Sierra de Aracena y Picos de Aroche
48	2024-05-09	ES	Campofrío. Fuente de las Tapias	29SQB1482	571	HSS087893		
49	2024-05-09	ES	El Campillo. Mirador del Odiel	29SQB0377	260	HSS087892		
50	2024-05-09	ES	Almonaster la Real. Barranco de los Rombos	29SQB7085	382	HSS087891		
51	2024-05-09	ES	Santa Ana la Real. La Serrezuela	29SPB9993	614	HSS087890		
52	2024-05-09	ES	Alajar. Puerto de Linares	29SQB0795	701	HSS087889		
53	2024-05-09	ES	Aracena. Mirador del Castañar	29SQB1399	684	HSS087888		
54	2024-05-09	ES	Jabugo. El Rojabugo	29SPC9900	535	HSS087887		
55	2024-05-09	ES	Cortegana. Cortijo de la Fuente de los Santos	29SPC8999	611	HSS087901		
56	2024-05-07	ES	Guillena. Dehesa de las Casetas	29SQB4876	296	HSS087874	12	Sierra Morena de Sevilla
57	2024-05-07	ES	El Ronquillo. Cerro Calamón	29SQB5082	390	HSS087933		
58	2024-05-07	ES	Almadén de la Plata. Arroyo de la Víbora	29SQB5797	444	HSS087931		
59	2024-05-07	ES	El Real de la Jara. Loma de los Lapares	29SQC6107	709	HSS087924		
60	2024-05-07	ES	Cazalla de la Sierra. Arroyo de Matajudíos	30STG5399	483	HSS087923		
61	2024-05-07	ES	Constantina. Canta el Gallo	30STG6693	544	HSS087920		
62	2024-05-07	ES	Alanís. La Ventilla	30STH6510	649	HSS087918		
63	2024-05-07	ES	Fuente del Arco. Cortijo de Santa Cruz	30STH4424	639	HSS087917		
64	2024-05-05	ES	Badajoz. Sagrajas	29SPD8210	179	HSS087667	13	Vegas Bajas
65	2024-05-09	ES	Alía. Collado de la Manrica	30SUJ0468	593	HSS087845	14	Villuercas
66	2024-05-09	ES	Carrascalejo. Collado de Arrebatacapas	30SUJ0590	912	HSS088136		
