# Supplementary material for: Lavandula stoechas subsp. luisieri (Rozeira) Rozeira: Variability of Chemical Composition of Essential Oil in Wild Populations
Source: Plants (Basel). 2025 Nov 10;14(22):3435. doi: 10.3390/plants14223435 (PMC12656282; doi:10.3390/plants14223435)
Supplement: Supplementary file 1 [file plants-14-03435-s001.zip › Suplement S2. Chemical Composition.pdf]

plants

**Table S2a.** Chemical composition of *L. stoechas* subsp. *luisieri* (Zone: 1. Alconera, 2. Atlantic coast, 3. Guadiana International, 4. Montes de Toledo).

| | | | 1 | 2 | | | | | | | 3 | | 4 | | | | | | | | | |
|---|---|---|---|---|---|---|---|---|---|---|---|---|---|---|---|---|---|---|---|---|---|---|
| **Components** | **RI-WAX** | **RI-HP5** | **1** | **2** | **3** | **4** | **5** | **6** | **7** | **8** | **9** | **10** | **11** | **12** | **13** | **14** | **15** | **16** | **17** | **18** | **19** | **20** |
| α-Pinene | 1025 | 933 | 1.65 | 1.16 | 1.01 | 1.28 | 1.45 | 1.28 | 1.19 | 0.67 | **1.96** | **1.72** | **3.62** | **4.54** | **3.22** | **2.40** | **5.36** | **2.98** | **3.38** | **2.01** | 1.92 | **2.89** |
| Methyl Butenol | 1043 | | 0.27 | 0.07 | 0.09 | 0.07 | 0.11 | 0.07 | 0.06 | 0.08 | 0.28 | 0.15 | 0.10 | 0.12 | 0.10 | 0.09 | 0.10 | 0.12 | 0.09 | 0.10 | 0.10 | 0.16 |
| α-Fenchene | 1060 | 948 | | | 0.02 | 0.02 | 0.05 | | | | | | 0.04 | 0.02 | 0.03 | 0.02 | 0.02 | 0.02 | 0.02 | | 0.01 | 0.02 |
| Camphene | 1069 | 953 | 0.08 | 0.08 | 0.12 | 0.10 | 0.16 | 0.09 | 0.10 | 0.05 | 0.06 | 0.07 | 0.12 | 0.12 | 0.12 | 0.15 | 0.22 | 0.10 | 0.17 | 0.09 | 0.13 | 0.16 |
| n-Hexanal | 1090 | 796 | | 0.02 | | | 0.02 | | | 0.04 | 0.02 | 0.02 | 0.02 | | | 0.01 | | | | | | |
| β-Pinene | 1114 | 978 | 0.41 | 0.16 | 0.58 | 0.39 | 0.28 | 0.09 | 0.03 | 0.03 | 0.39 | 0.38 | 0.19 | 0.30 | 0.25 | 0.28 | 0.27 | 0.14 | 0.25 | 0.24 | 0.27 | 0.01 |
| Sabinene | 1126 | 972 | 0.17 | 0.08 | 0.22 | 0.15 | 0.11 | 0.06 | 0.02 | | 0.16 | 0.12 | 0.11 | 0.13 | 0.10 | 0.12 | 0.12 | 0.06 | 0.10 | 0.09 | 0.12 | |
| Verbenene | 1130 | 971 | 0.05 | | 0.04 | 0.04 | 0.06 | 0.03 | 0.05 | 0.03 | 0.07 | 0.09 | 0.07 | 0.11 | 0.05 | 0.05 | 0.14 | 0.07 | 0.05 | 0.05 | 0.06 | 0.10 |
| Cymene Isomer | 1133 | 940 | **1.95** | 0.17 | **2.22** | **2.06** | **2.06** | 0.17 | **2.21** | 2.19 | **2.46** | **2.68** | **1.82** | 1.12 | | 1.37 | **1.96** | **2.36** | 0.17 | **2.28** | **2.31** | **2.71** |
| p-Xylene | 1146 | 865 | <0.01 | | | | | | 0.01 | | 0.01 | | | | | | | | | | | |
| δ-3-Carene | 1158 | 1009 | 0.09 | 0.06 | 0.01 | 0.01 | 0.03 | 0.03 | 0.07 | 0.05 | 0.04 | 0.03 | 1.53 | 0.50 | 0.31 | 0.48 | 0.28 | 0.55 | 0.12 | 0.30 | 0.33 | 1.62 |
| β-Myrcene | 1165 | 991 | 0.08 | 0.07 | 0.17 | 0.15 | 0.14 | 0.07 | 0.12 | 0.06 | 0.08 | 0.11 | 0.17 | 0.11 | 0.07 | 0.12 | 0.13 | 0.09 | 0.15 | 0.08 | 0.10 | 0.09 |
| 3-Methyl-2-Butenal | 1194 | 779 | 0.03 | 0.03 | 0.04 | 0.03 | 0.04 | 0.03 | 0.03 | 0.03 | 0.04 | 0.05 | 0.04 | 0.04 | 0.03 | 0.03 | 0.04 | 0.04 | 0.03 | 0.05 | 0.04 | 0.06 |
| Limonene | 1206 | 1021 | 0.18 | 0.23 | 0.35 | 0.40 | 0.61 | 0.16 | 0.14 | 0.18 | 0.20 | 0.22 | 0.59 | 0.44 | 0.39 | 0.39 | 0.49 | 0.39 | 0.39 | 0.23 | 0.27 | 0.28 |
| 1,8-Cineole | 1222 | 1039 | **24.13** | **8.82** | **21.80** | **14.36** | **13.06** | **5.67** | 1.20 | 1.05 | **18.60** | **18.21** | **4.43** | **14.01** | **11.79** | **7.56** | **10.70** | **4.60** | **10.50** | **8.25** | **11.96** | 0.18 |
| *cis*-β-Ocimene | 1238 | 1035 | 0.42 | 0.11 | 0.87 | 0.77 | 0.42 | 0.07 | 0.47 | 0.94 | 0.90 | 0.66 | 0.98 | 0.44 | 0.05 | 0.81 | 0.65 | 1.04 | 0.19 | 1.58 | 1.36 | 1.43 |
| γ-Terpinene | 1254 | 1058 | 0.07 | | 0.10 | 0.08 | 0.07 | | | | 0.05 | | 0.06 | | | | | | | | | |
| *trans*-β Ocimene | 1256 | 1046 | 0.05 | | 0.14 | 0.08 | 0.06 | | 0.09 | 0.07 | 0.04 | 0.05 | 0.06 | 0.03 | | 0.04 | 0.05 | 0.06 | | 0.09 | 0.07 | 0.09 |
| Prenyl Acetate | 1259 | 920 | 0.02 | 0.04 | 0.04 | 0.04 | 0.04 | 0.05 | 0.04 | 0.04 | 0.02 | 0.04 | 0.03 | 0.04 | 0.05 | 0.04 | 0.03 | 0.03 | 0.04 | 0.04 | 0.04 | 0.05 |
| *p*-Cymene | 1278 | 1025 | 0.13 | 0.16 | 0.14 | 0.12 | 0.17 | 0.14 | 0.08 | | 0.10 | 0.14 | 0.16 | 0.25 | 0.26 | 0.15 | 0.19 | 0.13 | 0.23 | 0.11 | 0.12 | 0.20 |
| *m*-Cymene | 1279 | 976 | <0.01 | | | | | | | | 0.01 | | 0.07 | 0.03 | | 0.03 | | 0.03 | | | 0.02 | 0.10 |
| α-Terpinolene | 1291 | 1086 | 0.05 | | 0.09 | 0.09 | 0.10 | | | 0.09 | 0.06 | 0.08 | 0.18 | 0.06 | | 0.07 | 0.10 | 0.12 | | 0.09 | 0.09 | 0.18 |
| Unknown I | | 1281 | | **1.98** | 1.31 | 1.47 | 1.30 | **2.01** | 1.67 | 1.62 | | 1.46 | 1.37 | 1.37 | **1.86** | **1.52** | 1.28 | 1.55 | 1.47 | 1.50 | 1.25 | 1.50 |

**Table S2a.** (Continued).

| | | | 1 | 2 | | | | | | | 3 | | 4 | | | | | | | | | |
|---|---|---|---|---|---|---|---|---|---|---|---|---|---|---|---|---|---|---|---|---|---|---|
| **Components** | **RI-WAX** | **RI-HP5** | **1** | **2** | **3** | **4** | **5** | **6** | **7** | **8** | **9** | **10** | **11** | **12** | **13** | **14** | **15** | **16** | **17** | **18** | **19** | **20** |
| 1,2,3,3-Tetramethyl-Cyclopenten-4-one | 1310 | | | | | | | 0.43 | | 0.46 | | 0.24 | 0.22 | 0.21 | | 0.17 | 0.19 | 0.29 | 0.16 | 0.24 | 0.20 | 0.32 |
| Prenol | 1322 | 772 | | 0.01 | 0.01 | 0.01 | | 0.01 | 0.01 | 0.01 | 0.01 | | | | 0.01 | | | | 0.02 | 0.02 | 0.02 | 0.02 |
| *cis*-3-Hexenyl Acetate | 1328 | 1008 | 0.01 | | | | | | | 0.02 | 0.02 | | | | | | | 0.02 | 0.02 | | 0.02 | |
| 6-Methyl-5-Hepten-2-one | 1351 | 986 | 0.01 | 0.02 | | 0.01 | | | | | 0.01 | 0.02 | 0.01 | 0.02 | 0.02 | 0.01 | | | 0.02 | 0.01 | 0.02 | 0.02 |
| *trans*-3-Hexenol | 1361 | 849 | | | | 0.09 | | | | | | | | 0.10 | | | | 0.09 | | | | |
| n-Hexanol | 1365 | 861 | | 0.03 | 0.02 | 0.02 | 0.03 | | 0.04 | 0.05 | | 0.02 | | 0.01 | | | | | | | | |
| Alloocimene | 1384 | 1129 | 0.03 | | | | | | | 0.03 | 0.04 | 0.03 | 0.05 | | | | | | | 0.07 | 0.04 | 0.07 |
| 1-Octen-3-yl Acetate | 1387 | 1086 | | 0.04 | 0.04 | | | 0.06 | 0.03 | 0.06 | | | | | | | | | | | | |
| *cis*-3-Hexenol | 1390 | 853 | 0.10 | 0.12 | 0.11 | 0.11 | 0.11 | 0.12 | 0.18 | 0.26 | 0.12 | 0.08 | 0.12 | 0.12 | | 0.04 | 0.13 | | 0.11 | 0.12 | 0.17 | 0.19 |
| n-Nonanal | 1406 | 1107 | | | | | | | | | | | | | | | | | | | | |
| *trans*-2-Hexenol | 1406 | 864 | | 0.02 | | 0.02 | | | 0.03 | 0.03 | | 0.04 | | 0.01 | | | 0.01 | 0.01 | | | 0.01 | |
| Rosefuran | 1412 | 1104 | 0.01 | | 0.01 | 0.02 | | | | 0.03 | 0.01 | | 0.03 | | | 0.03 | | 0.03 | | 0.03 | 0.02 | 0.03 |
| Fenchone | 1418 | 1090 | 0.45 | 1.40 | **4.41** | **5.31** | **12.92** | 0.30 | 0.23 | 1.22 | 0.04 | 0.38 | **7.39** | **4.88** | **3.98** | **2.75** | **4.13** | **4.61** | **3.15** | 0.78 | **2.32** | 2.06 |
| Unknown II | 1427 | 1083 | 0.75 | | | | | | | | 0.83 | | | | | | | | | | | |
| 1-Octen-3-ol | 1441 | 975 | | 0.30 | 0.16 | | 0.20 | 0.37 | 0.36 | 0.36 | | | 0.07 | | 0.08 | | 0.15 | | | | | |
| Acetic Acid | 1453 | 505 | | 0.30 | | | 0.03 | 0.27 | | | | | | 0.13 | 0.28 | 0.09 | 0.02 | | 0.20 | 0.03 | | |
| *cis*-Linalool Oxide (Furanoid) | 1460 | 1070 | 0.53 | 0.69 | 0.23 | 0.21 | 0.42 | 0.35 | 0.51 | 0.57 | 0.45 | 0.44 | 0.27 | 0.37 | 0.26 | 0.23 | 0.35 | 0.49 | 0.38 | 0.53 | 0.47 | 0.49 |
| Unknown III | 1471 | 1085 | | 1.05 | 0.66 | 0.69 | 0.66 | 1.15 | 0.94 | 0.92 | | 0.79 | 0.60 | 0.68 | 0.91 | 0.56 | 0.54 | 0.66 | 0.63 | 0.70 | 0.65 | 0.85 |
| $\alpha$-Cubebene | 1473 | 1351 | 0.01 | | | | | | | 0.06 | 0.01 | | | | | | | | | | | |
| *trans*-Linalool Oxide (Furanoid) | 1484 | 1086 | 0.36 | 0.51 | 0.13 | 0.16 | 0.34 | 0.24 | 0.40 | 0.45 | 0.41 | 0.36 | 0.21 | 0.27 | 0.17 | 0.17 | 0.25 | 0.36 | 0.27 | 0.43 | 0.40 | 0.38 |
| Cyclosativene | 1493 | 1363 | 0.19 | 0.34 | 0.10 | 0.17 | 0.12 | 0.21 | 0.48 | 0.38 | 0.16 | | 0.06 | | 0.08 | | | | | | | |
| $\alpha$-Campholenal | 1509 | 1125 | | | | 0.07 | 0.09 | | 0.09 | | 0.04 | 0.08 | 0.10 | 0.15 | 0.09 | 0.08 | 0.16 | 0.18 | | | 0.05 | 0.10 |
| $\alpha$-Copaene | 1511 | 1379 | 0.17 | 0.24 | 0.11 | 0.15 | 0.17 | 0.24 | 0.37 | 0.42 | 0.14 | | 0.13 | 0.07 | | | | 0.11 | | | 0.02 | |
| Camphor | 1541 | 1149 | 0.58 | 1.36 | **1.78** | 0.23 | 1.56 | **2.04** | **4.79** | 0.81 | 0.90 | 0.99 | 1.23 | 1.36 | **2.01** | 0.16 | **3.66** | **3.15** | **2.61** | 1.57 | **6.00** | **2.68** |
| Cyperene | 1541 | 1390 | 0.03 | | | | | | | | 0.04 | | | | | | | | | | | |

**Table S2a.** (Continued).

| | | | 1 | 2 | | | | | | | 3 | | 4 | | | | | | | | | |
|---|---|---|---|---|---|---|---|---|---|---|---|---|---|---|---|---|---|---|---|---|---|---|
| **Components** | **RI-WAX** | **RI-HP5** | **1** | **2** | **3** | **4** | **5** | **6** | **7** | **8** | **9** | **10** | **11** | **12** | **13** | **14** | **15** | **16** | **17** | **18** | **19** | **20** |
| α-Gurjunene | 1548 | 1407 | | | | | | | 0.09 | | | | | | | | | | | | | |
| *cis*-Sabinene Hydrate | 1550 | 1105 | | | 0.05 | 0.05 | | | | | | | | | | | | | | | | |
| Unknown IV | | 1551 | | 0.22 | 0.70 | 0.46 | 0.67 | 0.27 | 0.49 | 0.56 | | 0.48 | 0.52 | 0.46 | 0.15 | 0.53 | 0.51 | 0.40 | 0.45 | 1.42 | 1.16 | 1.22 |
| Unknown V | 1552 | | | 0.37 | 0.22 | 0.23 | 0.24 | 0.43 | 0.35 | 0.39 | | 0.28 | 0.26 | 0.24 | 0.37 | 0.22 | 0.23 | 0.28 | 0.26 | 0.29 | 0.21 | 0.32 |
| Linalool | 1553 | 1100 | **3.01** | **3.14** | 1.09 | 1.26 | **2.10** | **1.89** | **2.87** | **3.33** | **2.41** | **1.96** | **1.87** | **2.14** | 1.31 | **1.72** | **2.53** | **2.74** | **2.65** | **3.29** | **2.87** | **2.80** |
| Linalyl Acetate | 1558 | 1255 | 0.06 | 0.14 | 0.07 | 0.06 | 0.09 | | 0.04 | 0.09 | 0.05 | 0.06 | | 0.07 | | 0.05 | 0.09 | 0.05 | 0.11 | 0.09 | 0.09 | 0.22 |
| Unknown VI | 1575 | 1156 | | 0.33 | 0.44 | 0.46 | 0.44 | 0.32 | 0.61 | 0.74 | | 0.28 | 0.43 | 0.36 | 0.18 | | 0.38 | 0.54 | 0.18 | 0.50 | 0.42 | 0.56 |
| Fenchol<*exo*> | 1582 | 1112 | | 0.02 | 0.20 | 0.17 | 0.24 | | | | | | 0.09 | | 0.03 | 0.03 | 0.07 | | | | | |
| Pinocarvone | 1589 | 1164 | 0.14 | 0.07 | 0.12 | 0.10 | 0.10 | | | | 0.11 | | 0.09 | 0.12 | 0.09 | | 0.16 | | 0.08 | 0.08 | 0.09 | 0.11 |
| γ-Amorphene | 1592 | 1490 | 0.01 | | | | | | | | 0.03 | | | | | 0.13 | | | | | | |
| Bornyl Acetate | 1599 | 1288 | 0.28 | 0.11 | 0.14 | 0.13 | 0.21 | 0.11 | 0.11 | | | 0.12 | 0.16 | 0.18 | 0.15 | 0.54 | 0.17 | 0.14 | 0.14 | 0.11 | 0.16 | 0.11 |
| *trans*-α-Necrodyl Acetate | 1606 | 1280 | **11.11** | **20.75** | **17.10** | **16.59** | **15.68** | **21.69** | **15.30** | **16.05** | **20.18** | **20.28** | **19.94** | **20.16** | **24.59** | **29.94** | **18.17** | **20.88** | **22.38** | **24.46** | **26.04** | **21.41** |
| Hotrienol | 1609 | 1108 | | | | | | | | | | | | | | | | | | | | |
| Unknown VII | 1609 | 1326 | 0.48 | | | | | | | | 0.51 | | | | | | | | | | | |
| *trans*-β Caryophyllene | 1617 | 1450 | 0.28 | 0.17 | 0.10 | 0.18 | 0.36 | 0.19 | 0.62 | 0.70 | 0.16 | 0.11 | 0.25 | 0.11 | | 0.12 | 0.07 | 0.20 | 0.05 | 0.16 | 0.14 | 0.21 |
| Lavandulyl Acetate | 1619 | 1284 | **4.43** | **6.22** | **4.24** | **4.17** | **4.15** | **6.34** | **4.83** | **5.54** | **5.28** | **4.13** | **5.01** | **3.87** | **5.83** | **3.92** | **3.97** | **4.52** | **4.81** | **4.97** | **2.95** | **4.65** |
| Terpinen-4-ol | 1622 | 1184 | 0.19 | 0.12 | 0.28 | 0.21 | 0.17 | | 0.04 | | 0.10 | 0.16 | 0.17 | 0.22 | 0.19 | 0.16 | 0.21 | 0.09 | 0.17 | 0.09 | 0.12 | 0.12 |
| *cis*-α-Necrodyl Acetate | 1629 | 1302 | 1.27 | **1.81** | 1.29 | 0.95 | 0.82 | 1.72 | 1.40 | 1.16 | 1.59 | 1.25 | 1.31 | 1.10 | 1.35 | 1.21 | 1.39 | 0.94 | **1.72** | 1.09 | 1.66 | 1.76 |
| Arbozol | 1636 | 1296 | 1.11 | 1.71 | **1.70** | 1.55 | **1.58** | 1.25 | 1.40 | 1.54 | **2.13** | **1.70** | 0.84 | **1.65** | 1.24 | **1.64** | 1.54 | 1.50 | 1.16 | 1.65 | 1.96 | **2.36** |
| Valerena-4,7(11)-diene | 1637 | 1455 | | | | | | | | | | | | | | | | | | | | |
| Sabina Ketone | 1645 | 1157 | 0.03 | | 0.03 | 0.02 | | | | | 0.03 | 0.03 | | 0.02 | | | | | | | | |
| Benzeneacetaldehyde | 1649 | 1049 | 0.01 | | | | | 0.04 | | | 0.03 | | | | | | | | | | | |
| Myrtenal | 1651 | 1197 | 0.11 | | | 0.14 | | | | | 0.11 | | | | | | 0.19 | | | | | |
| Unknown VIII | 1654 | 1326 | | 0.64 | 0.46 | 0.64 | 0.43 | 0.64 | 0.65 | 0.48 | | 0.68 | 0.51 | 0.50 | 0.65 | 0.60 | 0.46 | 0.54 | 0.56 | 0.57 | 0.51 | 0.54 |
| *cis*-Verbenol | 1660 | 1141 | | | | | | | | | | | | | | | 0.20 | | | | | |

**Table S2a.** (Continued).

| | | | 1 | 2 | | | | | | | 3 | | 4 | | | | | | | | | |
|---|---|---|---|---|---|---|---|---|---|---|---|---|---|---|---|---|---|---|---|---|---|---|
| **Components** | **RI-WAX** | **RI-HP5** | **1** | **2** | **3** | **4** | **5** | **6** | **7** | **8** | **9** | **10** | **11** | **12** | **13** | **14** | **15** | **16** | **17** | **18** | **19** | **20** |
| *trans*-Muurola-3,5-diene | 1661 | 1453 | 0.09 | | | | | | | | | 0.12 | | | | | | 0.04 | | | 0.13 | 0.14 |
| Alloaromadendrene | 1664 | 1471 | 0.16 | 0.28 | 0.16 | | 0.19 | 0.37 | 0.33 | 0.33 | 0.16 | | | | | | | | | | | |
| 5-Methylene-2,3,4,4-tetrame-2-Cyclopentenone | 1668 | 1187 | **2.55** | **2.86** | **1.83** | **1.89** | **2.23** | **3.06** | **4.00** | **2.63** | **2.43** | **2.09** | 1.57 | **1.70** | **1.55** | 1.26 | 1.63 | 1.78 | 1.33 | 1.59 | 1.77 | **2.44** |
| Lavandulol | 1669 | 1165 | | 0.14 | 0.19 | 0.30 | 0.08 | 0.39 | 0.32 | 0.55 | 0.25 | 0.21 | 0.36 | 0.27 | 0.23 | 0.23 | 0.21 | 0.53 | 0.27 | 0.40 | 0.21 | 0.20 |
| Unknown IX | 1673 | | | 0.75 | 0.49 | 0.66 | 0.50 | 0.77 | | 0.57 | | 0.69 | 0.66 | 0.73 | 0.78 | | 0.28 | 0.78 | 0.68 | 0.73 | 0.67 | 0.50 |
| *trans*-α-Necrodol | 1679 | 1172 | **7.84** | **6.33** | **7.38** | **9.67** | **5.43** | **11.23** | **4.59** | **12.38** | **6.96** | **6.22** | **11.52** | **8.27** | **8.53** | **10.48** | **7.89** | **12.26** | **8.85** | **13.57** | **7.70** | **8.51** |
| *trans*-Cadina-1(6),4-diene | 1682 | 1477 | 0.03 | | 0.07 | 0.08 | 0.07 | | 0.15 | 0.14 | 0.06 | | 0.06 | | | | 0.15 | 0.06 | | | | |
| *trans*-Verbenol | 1692 | 1151 | | 0.19 | 0.08 | 0.30 | 0.21 | 0.48 | 0.19 | 0.27 | | 0.19 | 0.63 | 0.77 | 0.56 | 0.35 | 0.95 | 0.66 | 0.59 | 0.40 | 0.37 | 0.36 |
| α-Humulene | 1694 | 1454 | | | | | | | | 0.10 | 0.04 | | | | | | | | | | | |
| β-Acoradiene | 1694 | 1466 | | | | | | | | | | 0.06 | | 0.05 | | 0.05 | | | 0.10 | | | |
| α-Terpineol | 1696 | 1195 | 0.45 | 0.21 | 0.59 | 0.40 | 0.30 | 0.11 | | 0.05 | 0.38 | 0.38 | 0.22 | 0.34 | 0.36 | 0.32 | 0.35 | 0.24 | 0.31 | 0.31 | 0.31 | 0.12 |
| γ-Muurolene | 1700 | 1485 | | | | | 0.03 | | 0.06 | 0.06 | | | | | | | | 0.01 | | | 0.04 | |
| Heptadecane | 1700 | 1700 | | | 0.07 | 0.06 | | | 0.04 | | | 0.07 | | 0.03 | | | | 0.03 | | 0.10 | 0.06 | |
| Myrtenyl Acetate | 1702 | 1329 | | 0.31 | 0.40 | 0.54 | 0.26 | 0.33 | 0.18 | | 0.46 | 0.47 | 0.35 | 0.27 | 0.23 | 0.13 | 0.30 | 0.07 | 0.34 | 0.38 | 0.21 | 0.51 |
| Ledene | 1708 | 1485 | | | | | | | 0.09 | 0.07 | | | | | | | | | | | | |
| α-Amorphene | 1711 | 1506 | | | | | | | 0.36 | | | | | | | | | | | | | |
| Borneol | 1713 | 1167 | | | | | | | | | | | | | | 0.12 | | | | | | |
| Neryl Acetate | 1723 | 1361 | 0.11 | 0.56 | 0.27 | 0.26 | 0.19 | 0.37 | 0.65 | 0.52 | 0.18 | 0.29 | 0.25 | 0.16 | 0.24 | 0.28 | 0.20 | 0.42 | 0.38 | 0.17 | 0.30 | 0.16 |
| 10-Epizonarene | 1725 | 1506 | | | | | | | | | | | | | | | | | | | | |
| *cis*-Muurola-4(14),5-diene | 1735 | 1466 | | | | 0.15 | | | 0.12 | 0.18 | | 0.24 | 0.10 | 0.17 | | 0.20 | 0.15 | 0.04 | | 0.10 | 0.18 | 0.15 |
| *cis*-α-Necrodol | 1735 | 1150 | 1.46 | **1.85** | **1.47** | **1.77** | 1.46 | **2.22** | 1.99 | **2.29** | 1.46 | 1.36 | **1.75** | 1.39 | **1.55** | 1.32 | 1.32 | **2.12** | 1.27 | **1.93** | 1.34 | 1.62 |
| Eremophilene | 1735 | 1486 | | | | | | | | | | | | 0.23 | | | | | | | | |
| Valencene | 1736 | 1409 | | | | | | | | | | | | | | | | | | | 0.11 | 0.10 |
| Verbenone | 1736 | 1208 | | 0.23 | 0.12 | 0.13 | 0.24 | 0.24 | 0.21 | | 0.30 | 0.34 | 0.40 | 0.54 | 0.52 | 0.21 | 0.50 | 0.26 | 0.43 | | 0.25 | 0.50 |
| α-Selinene | 1737 | 1501 | | 0.19 | 0.10 | 0.08 | 0.17 | 0.17 | 0.16 | 0.19 | | 0.10 | 0.10 | 0.10 | 0.08 | 0.16 | 0.10 | 0.08 | 0.13 | 0.35 | 0.27 | 0.19 |

**Table S2a.** (Continued).

| | | | 1 | 2 | | | | | | | 3 | | 4 | | | | | | | | | |
|---|---|---|---|---|---|---|---|---|---|---|---|---|---|---|---|---|---|---|---|---|---|---|
| **Components** | **RI-WAX** | **RI-HP5** | **1** | **2** | **3** | **4** | **5** | **6** | **7** | **8** | **9** | **10** | **11** | **12** | **13** | **14** | **15** | **16** | **17** | **18** | **19** | **20** |
| Unknown X | 1739 | 1173 | **1.98** | | | | | | | | 1.73 | | | | | | | | | | | |
| $\alpha$-Muurolene | 1746 | 1501 | 0.18 | | | | | | | 0.06 | 0.20 | | | | | | | | | | | |
| β-Selinene | 1746 | 1502 | 0.17 | 0.36 | 0.28 | 0.18 | 0.35 | 0.38 | 0.30 | 0.34 | 0.15 | 0.20 | 0.25 | 0.25 | 0.20 | 0.21 | 0.31 | 0.17 | 0.30 | 0.44 | 0.40 | 0.42 |
| Geranyl Acetate | 1758 | 1380 | 0.16 | 0.20 | 0.86 | 0.29 | 0.45 | 0.21 | 0.12 | 0.09 | 0.17 | 0.29 | 0.18 | 0.26 | 0.22 | 0.28 | 0.26 | 0.19 | 0.27 | 0.20 | 0.34 | 0.46 |
| (E,E) $\alpha$ Famesene | 1760 | 1508 | | | | | | | | | | | | | | | | | | | | |
| Carvone | 1762 | 1246 | 0.04 | | | 0.06 | 0.11 | | 0.03 | | 0.03 | 0.04 | | 0.08 | | 0.06 | | 0.07 | | | 0.05 | |
| *trans*-Linalool Oxide (Pyranoid) | 1767 | 1170 | | | | | | | 0.03 | | 0.02 | 0.04 | | 0.02 | | 0.02 | | 0.03 | | | 0.04 | |
| δ-Cadinene | 1776 | 1524 | 0.18 | 0.38 | 0.47 | 0.57 | 0.61 | 0.37 | 1.34 | 1.49 | 0.43 | 0.24 | 0.55 | 0.28 | 0.13 | 0.11 | | 0.39 | | 0.23 | 0.18 | 0.51 |
| Unknown XI | 1793 | 1173 | | 1.22 | 1.30 | **1.67** | 1.28 | 1.13 | 1.91 | **2.31** | | 1.20 | 1.73 | 0.97 | 0.57 | 0.98 | 1.11 | 1.88 | 0.64 | **1.71** | 1.00 | 1.33 |
| γ-Cadinene | 1782 | 1515 | | 0.58 | 0.20 | 0.03 | 0.26 | 0.62 | 0.65 | 0.71 | | 0.19 | 0.12 | 0.15 | 0.16 | 0.13 | 0.27 | 0.23 | 0.32 | | 0.18 | 0.20 |
| 1,7,7-Trimethylbicyclo[2.2.1]hept-5-en-2-ol | 1785 | 1120 | | | | | | | | 0.03 | | | 0.13 | | 0.11 | | 0.21 | | | | | 0.15 |
| Myrtenol | 1798 | 1202 | | | | | | | | | 0.08 | | | | | | | | | | | |
| *p*-Methyl Acetophenone | 1800 | 1183 | | | | | | | | | | | | | | | | | | | | 0.07 |
| Eudesma-3,7(11)-diene | 1805 | 1532 | 0.65 | 0.60 | 1.31 | 0.82 | 1.42 | 0.85 | 1.41 | 1.15 | 0.62 | 1.29 | 0.84 | 1.31 | 0.40 | 0.99 | 1.17 | 0.70 | 0.88 | **2.69** | **2.01** | 2.11 |
| $\alpha$-Cadinene | 1817 | 1538 | | | | | | | | | | | 0.08 | 0.10 | | 0.10 | 0.17 | | 0.20 | | 0.11 | |
| *trans*-Carveol | 1822 | 1223 | | | | | 0.09 | | | | | | | | | | | | | | | |
| Phenethyl Acetate | 1828 | 1257 | | | 0.11 | | | 0.06 | | | | | | | | | | | | | | |
| *p*-Cymen-8-ol | 1839 | 1189 | 0.02 | | | 0.03 | | 0.11 | | | | 0.04 | 0.23 | 0.12 | 0.13 | 0.09 | | 0.10 | 0.39 | | 0.07 | 0.44 |
| *cis*-Carveol | 1852 | 1232 | | | | 0.07 | | | 0.10 | | 0.06 | 0.08 | 0.09 | 0.12 | 0.10 | 0.07 | 0.14 | 0.08 | 0.09 | | 0.06 | 0.10 |
| Unknown XII | 1852 | 1393 | | | | | | | | | | | | | | | | | | | | |
| Germacrene B | 1853 | 1557 | | | | | | | | | | | | | | | | | | | | |
| *trans*-Calamenene | 1857 | 1527 | | 0.35 | | 0.08 | | | 0.07 | | | 0.12 | | 0.17 | | 0.16 | 0.14 | 0.12 | 0.22 | 0.13 | 0.10 | |
| Unknown XIII | | 1860 | | 1.43 | 1.46 | **1.84** | **1.78** | 1.88 | **3.60** | **3.42** | | 0.54 | 1.71 | 0.58 | 0.56 | 0.17 | 0.14 | 1.55 | | | | 0.41 |
| Unknown XIV | | 1861 | **1.93** | | | | | | | | **2.04** | | | | | | | | | | | |
| *meta*-Cymen-8-ol | 1861 | 1183 | | | | 0.19 | | | 0.20 | | | 0.24 | | 0.10 | | 0.09 | | 0.18 | | | 0.21 | |

**Table S2a.** (Continued).

| Components | RI-WAX | RI-HP5 | 1 | 2 | | | | | | | 3 | | 4 | | | | | | | | | |
|---|---|---|---|---|---|---|---|---|---|---|---|---|---|---|---|---|---|---|---|---|---|---|
| | | | **1** | **2** | **3** | **4** | **5** | **6** | **7** | **8** | **9** | **10** | **11** | **12** | **13** | **14** | **15** | **16** | **17** | **18** | **19** | **20** |
| Unknown XV | 1862 | 1393 | | | | 0.68 | | | 0.85 | | | 0.94 | | 0.26 | | 0.43 | | 0.70 | | | 0.79 | |
| Unknown XVI | | 1870 | 1.50 | 1.10 | 1.12 | 1.25 | 1.34 | 1.55 | **2.75** | **2.71** | 1.48 | 0.40 | 1.31 | 0.41 | 0.47 | 0.11 | 0.09 | 1.15 | | | | 0.28 |
| Cubebol Isomer I | 1903 | 1516 | 0.43 | | | | | | 0.66 | | 0.28 | | | | | | | 0.44 | | | 0.40 | |
| *epi*-Cubebol | 1903 | 1498 | 0.64 | 0.81 | 0.21 | 0.55 | 0.28 | 0.87 | 0.75 | 0.77 | 0.25 | 0.09 | 0.28 | 0.15 | 0.12 | 0.06 | | 0.26 | | 0.55 | | |
| 10,11-Epoxycalamenene | 1903 | 1498 | | | | | | | | | 0.05 | | | | | | | | | | | |
| *cis*-Muurol-5-en-4-β-ol | 1905 | 1548 | | | | | | | | | | | | | | | | | | | | |
| Unknown XVII | 1946 | 1564 | 0.47 | | | | | | | | 0.47 | | | | | 0.07 | | | | | | |
| α-Calacorene | 1944 | 1542 | | | 0.08 | 0.09 | | | 0.13 | 0.16 | | 0.07 | 0.07 | 0.07 | | 0.04 | 0.15 | 0.08 | | | 0.05 | 0.11 |
| Palustrol | 1958 | 1568 | 0.05 | | | 0.04 | | | 0.10 | 0.09 | 0.05 | 0.02 | | 0.02 | | | | 0.03 | | | | |
| *trans*-Muurol-5-en-4-β-ol | 1958 | | | 0.52 | 0.15 | 0.42 | 0.22 | 0.47 | 0.65 | 0.77 | | 0.05 | 0.26 | 0.07 | | | | 0.22 | | | | |
| n-Dodecanol | 1975 | 1476 | | | | | | | | | | | | | | | | | | | | |
| Unknown XVIII | 1996 | 1564 | | 0.82 | 0.42 | 0.51 | 0.48 | 0.77 | 0.90 | 1.00 | | 0.16 | 0.51 | 0.21 | 0.35 | | | 0.48 | | | | 0.11 |
| Caryophyllene Oxide | 2014 | 1578 | 0.34 | 0.69 | 0.21 | 0.27 | 0.31 | 0.69 | 0.66 | 0.62 | 0.26 | 0.11 | 0.22 | 0.15 | 0.30 | 0.04 | | 0.26 | | | 0.06 | 0.16 |
| Unknown XIX | 2059 | 1578 | 0.61 | | | | | | | | 0.67 | | | | | | | | | | | |
| Ledol | 2066 | 1574 | 0.35 | 0.74 | 0.31 | 0.28 | 0.39 | 0.75 | 0.83 | 0.76 | 0.34 | 0.38 | 0.27 | 0.08 | 0.32 | 0.06 | | 0.29 | | | 0.03 | 0.15 |
| 1,10-Di-*epi*-Cubenol | 2099 | 1614 | | | 0.12 | 0.29 | | | 0.20 | 0.10 | | 0.45 | 0.17 | 0.33 | 0.28 | 0.46 | 0.54 | 0.24 | 0.73 | 0.11 | 0.33 | 0.30 |
| Viridiflorol | 2103 | 1610 | **1.75** | **2.67** | 1.12 | 1.42 | 1.45 | **2.76** | **3.22** | **2.90** | 1.66 | 0.56 | 1.01 | 0.62 | 0.92 | 0.14 | 0.10 | 1.05 | | | 0.01 | 0.44 |
| 1-*epi*-Cubenol | 2107 | 1613 | 0.04 | 0.48 | 0.23 | 0.28 | 0.29 | 0.41 | 0.56 | 0.58 | 0.36 | 0.11 | 0.20 | 0.12 | 0.19 | 0.04 | | | | | | 0.09 |
| Unknown XX | 2115 | 1577 | | 0.92 | 0.51 | 0.61 | 0.60 | 0.99 | 1.38 | 1.99 | | 0.18 | 0.66 | 0.05 | 0.31 | 0.09 | | 0.56 | | | 0.10 | 0.27 |
| Spathulenol | 2149 | 1572 | | | | | | | | | 0.02 | | | | | | | | | | | |
| Copaborneol | 2199 | 1613 | 0.83 | 1.37 | 0.54 | 0.70 | 0.71 | 1.43 | 1.69 | 1.55 | 0.93 | 0.19 | 0.48 | 0.31 | 0.45 | | | 0.50 | | | 0.04 | |
| (E)-1-(6,10-Dimethylundec-5-en-2-yl)-4-Methylbenzene | 2212 | | | | | | | | 0.40 | | 0.12 | 0.06 | | | | | | | | | | |
| Pentylcurcumene | 2212 | | | | 0.45 | 0.24 | 0.16 | | | 0.31 | | | 0.18 | | | | | | | | | |
| T-Muurolol | 2215 | 1645 | 0.04 | 0.35 | 0.22 | 0.30 | 0.16 | 0.37 | 0.36 | 0.32 | 0.25 | 0.12 | 0.27 | 0.12 | 0.16 | | | 0.27 | | | 0.13 | |
| α-Muurolol | 2224 | 1649 | 0.10 | | | 0.05 | 0.05 | | | | 0.10 | 0.03 | | 0.02 | | | | 0.11 | | | 0.07 | |

**Table S2a.** (Continued).

| | | | 1 | 2 | | | | | | | 3 | | 4 | | | | | | | | | |
|---|---|---|---|---|---|---|---|---|---|---|---|---|---|---|---|---|---|---|---|---|---|---|
| **Components** | **RI-WAX** | **RI-HP5** | **1** | **2** | **3** | **4** | **5** | **6** | **7** | **8** | **9** | **10** | **11** | **12** | **13** | **14** | **15** | **16** | **17** | **18** | **19** | **20** |
| Cadalene | 2241 | 1670 | 0.11 | 0.14 | 0.05 | 0.04 | 0.07 | 0.14 | 0.12 | 0.11 | 0.04 | 0.12 | 0.06 | 0.08 | 0.11 | 0.08 | 0.12 | 0.06 | 0.13 | | 0.09 | 0.15 |
| α-Cadinol | 2249 | 1652 | | | 0.43 | 0.88 | 0.31 | | 0.26 | | | **6.60** | **3.01** | **4.77** | **2.99** | **6.09** | **8.24** | 1.70 | **10.78** | 0.25 | **5.55** | **4.85** |
| β-Eudesmol | 2251 | 1656 | | | | | 0.13 | | | | | | | | | | | | | | | |
| *neo*-Intermedeol | 2264 | 1661 | | | | | | | | | | | | | | | | | | | | |
| (E)-1-(6,10-Dimethylundec-5,9-dien-2-yl)-4-Methylbenzene | 2293 | | | | | | | | | | | | | | | | | | | | | |
| Pentenylcurcumene | 2293 | 1970 | | 0.12 | 0.07 | 0.08 | | 0.07 | | | | | | | | | | | | | | |
| Eudesm-7(11)-en-4-ol | 2312 | 1696 | 0.15 | | 0.23 | 0.18 | 0.21 | 0.17 | 0.17 | 0.21 | 0.10 | 0.14 | 0.17 | 0.19 | | 0.30 | 0.27 | 0.20 | 0.20 | 0.60 | 0.45 | 0.46 |
| *cis*-14-nor-Muurol-5-en-4-one | 2329 | 1696 | | | | 0.07 | | | | | | 0.71 | 0.28 | 0.45 | 0.40 | 0.42 | 0.63 | 0.15 | 0.88 | 0.17 | 0.45 | 0.59 |
| Vetiveryl Acetate Isomer | 2342 | 1872 | | | | | | | | | | | | | | | | | | 0.36 | | 0.28 |
| α-Cyperone | 2370 | 1706 | | | | | | | | | | | | | | | | | | | 0.12 | |
| Phenethyl Phenylacetate | 2817 | 1915 | | | | | | | | | | | | | | | | | | | | |
| **% Identification** | | | 80.99 | 85.24 | 91.04 | 87.70 | 89.14 | 87.49 | 84.68 | 88.82 | 87.14 | 90.10 | 90.99 | 91.37 | 87.28 | 87.83 | 90.15 | 91.12 | 90.35 | 87.95 | 96.70 | 85.37 |
| **Yield (g/kg)** | | | 2.65 | 2.32 | 4.43 | 3.78 | 3.57 | 1.48 | 1.42 | 1.70 | 3.46 | 4.97 | 4.75 | 4.65 | 5.65 | 8.17 | 5.53 | 4.83 | 5.43 | 4.65 | 5.35 | 3.01 |
| Monoterpene hydrocarbons | | | 5.41 | 2.28 | 6.08 | 5.74 | 5.77 | 2.19 | 4.57 | 4.39 | 6.62 | 6.38 | 9.82 | 8.20 | 4.85 | 6.48 | 9.98 | 8.14 | 5.22 | 7.31 | 7.22 | 9.95 |
| Oxygen-containing monoterpenes | | | 56.74 | 55.22 | 64.34 | 58.27 | 60.77 | 56.10 | 40.45 | 46.54 | 60.63 | 59.32 | 58.69 | 62.06 | 65.02 | 63.02 | 59.36 | 60.14 | 63.04 | 63.30 | 67.11 | 50.82 |
| Sesquiterpene hydrocarbons | | | 2.26 | 3.63 | 3.03 | 2.62 | 3.82 | 3.54 | 6.85 | 6.65 | 2.24 | 2.86 | 2.67 | 3.14 | 1.16 | 2.48 | 2.80 | 2.29 | 2.33 | 4.10 | 4.01 | 4.29 |
| Oxygen-containing sesquiterpenes | | | 4.72 | 7.63 | 3.77 | 5.66 | 4.51 | 7.92 | 10.11 | 8.67 | 4.65 | 8.85 | 6.34 | 6.95 | 5.73 | 7.19 | 9.15 | 5.57 | 11.71 | 1.87 | 7.19 | 6.73 |
| Unknown | | | 4.14 | 5.65 | 4.73 | 4.24 | 4.55 | 5.83 | 6.60 | 5.86 | 5.27 | 4.61 | 3.20 | 4.20 | 3.36 | 3.38 | 3.84 | 3.91 | 3.18 | 3.95 | 4.41 | 5.69 |
| Other | | | 7.72 | 10.83 | 9.09 | 11.17 | 9.72 | 11.91 | 16.10 | 16.71 | 7.73 | 8.08 | 10.27 | 6.82 | 7.16 | 5.28 | 5.02 | 11.07 | 4.87 | 7.42 | 6.76 | 7.89 |

**Table S2b.** Chemical composition of *L. stoechas* subsp. *luisieri* (Zone: 5. Rio Tejo, 6. Serra da Gardunha, 7. Serra de Alvelos, 8. Serra de Monchique).

| | | | **5** | | **6** | | | | | **7** | | | | | **8** | | | | | | |
|---|---|---|---|---|---|---|---|---|---|---|---|---|---|---|---|---|---|---|---|---|---|
| **Components** | **RI-WAX** | **RI-HP5** | **21** | **22** | **23** | **24** | **25** | **26** | **27** | **28** | **29** | **30** | **31** | **32** | **33** | **34** | **35** | **36** | **37** | **38** | **39** |
| α-Pinene | 1025 | 933 | 1.47 | 1.50 | 1.42 | 0.57 | 1.53 | 1.90 | 1.43 | 1.07 | 0.41 | **4.77** | 0.68 | 1.04 | 0.87 | 1.50 | 0.58 | 1.19 | 0.74 | 0.93 | 1.18 |
| Methyl Butenol | 1043 | | 0.09 | 0.08 | 0.11 | 0.19 | 0.06 | 0.08 | 0.06 | 0.02 | 0.05 | 0.11 | 0.05 | 0.07 | 0.12 | 0.11 | 0.07 | 0.09 | 0.14 | 0.07 | 0.07 |
| α-Fenchene | 1060 | 948 | | | 0.01 | | 0.02 | | | | | | | | | 0.05 | | | | | |
| Camphene | 1069 | 953 | 0.05 | 0.06 | 0.10 | 0.04 | 0.09 | 0.13 | 0.08 | 0.04 | 0.09 | 0.35 | 0.04 | 0.05 | 0.05 | 0.15 | 0.05 | 0.10 | 0.12 | 0.15 | 0.10 |
| n-Hexanal | 1090 | 796 | 0.01 | | | 0.01 | 0.02 | | | 0.01 | | 0.03 | | 0.03 | | | 0.02 | | 0.01 | 0.02 | 0.03 |
| β-Pinene | 1114 | 978 | 0.34 | 0.04 | 0.13 | 0.04 | 0.21 | 0.13 | 0.19 | 0.08 | 0.20 | 0.06 | 0.01 | 0.10 | 0.19 | 0.28 | 0.01 | 0.05 | 0.11 | 0.19 | 0.17 |
| Sabinene | 1126 | 972 | 0.13 | | 0.05 | | 0.07 | 0.03 | 0.04 | | | 0.04 | | | 0.09 | 0.11 | | | 0.04 | 0.07 | 0.09 |
| Verbenene | 1130 | 971 | 0.04 | 0.03 | 0.06 | 0.04 | 0.05 | 0.09 | 0.05 | 0.06 | | 0.12 | 0.04 | 0.05 | 0.03 | 0.06 | 0.02 | 0.04 | 0.03 | 0.03 | 0.03 |
| Cymene Isomer | 1133 | 940 | **2.78** | 0.30 | **2.42** | **2.51** | 0.69 | **2.41** | 1.48 | **1.78** | 0.09 | **1.91** | **2.54** | **2.44** | **3.16** | **2.04** | **2.05** | **2.09** | **1.85** | 1.85 | 0.42 |
| p-Xylene | 1146 | 865 | <0,01 | | 0.01 | | | | | | | 0.01 | <0,01 | | | 0.01 | | | 0.01 | 0.01 | |
| δ-3-Carene | 1158 | 1009 | 0.19 | 0.27 | 0.36 | 0.09 | 0.13 | 0.21 | 0.36 | 0.17 | 0.08 | 0.05 | 0.09 | 0.22 | 0.04 | 0.03 | 0.04 | 0.07 | 0.04 | 0.10 | 0.08 |
| β-Myrcene | 1165 | 991 | 0.08 | 0.06 | 0.06 | 0.04 | 0.08 | 0.04 | 0.06 | 0.04 | 0.03 | 0.07 | 0.02 | 0.09 | 0.08 | 0.13 | 0.06 | 0.06 | 0.04 | 0.07 | 0.06 |
| 3-Methyl-2-Butenal | 1194 | 779 | 0.04 | 0.03 | 0.04 | 0.04 | 0.03 | 0.04 | 0.02 | 0.02 | 0.02 | 0.04 | 0.03 | 0.04 | 0.05 | 0.04 | 0.03 | 0.04 | 0.03 | 0.03 | 0.03 |
| Limonene | 1206 | 1021 | 0.18 | 0.25 | 0.26 | 0.16 | 0.35 | 0.16 | 0.24 | 0.18 | 0.15 | 0.35 | 0.09 | 0.18 | 0.14 | 0.53 | 0.14 | 0.18 | 0.12 | 0.16 | 0.23 |
| 1,8-Cineole | 1222 | 1039 | **9.88** | 0.26 | **2.39** | 0.41 | **4.37** | **3.32** | **2.20** | 0.60 | 1.25 | 1.33 | 0.20 | 0.70 | **5.38** | **12.86** | 0.09 | 1.04 | **5.96** | **12.42** | **6.75** |
| *cis*-β-Ocimene | 1238 | 1035 | 1.06 | 0.36 | 0.91 | 0.89 | 0.47 | 0.78 | 1.01 | 0.73 | 0.03 | 0.22 | 0.74 | 0.71 | 1.19 | 0.42 | 0.96 | 0.72 | 1.08 | 0.97 | 0.16 |
| γ-Terpinene | 1254 | 1058 | 0.06 | | | 0.07 | | | | 0.06 | | | | | | 0.06 | | | 0.06 | | |
| *trans*-β Ocimene | 1256 | 1046 | 0.06 | | 0.06 | 0.02 | | | 0.06 | 0.10 | | | 0.04 | | | 0.06 | 0.08 | 0.10 | 0.13 | 0.10 | |
| Prenyl Acetate | 1259 | 920 | 0.04 | 0.04 | 0.04 | 0.03 | 0.04 | 0.05 | 0.02 | 0.02 | 0.05 | 0.06 | 0.05 | 0.05 | 0.03 | 0.03 | 0.04 | 0.04 | 0.02 | 0.03 | 0.04 |
| *p*-Cymene | 1278 | 1025 | 0.09 | 0.12 | 0.12 | 0.03 | 0.15 | 0.15 | 0.10 | 0.05 | 0.10 | 0.21 | 0.05 | 0.09 | 0.05 | 0.16 | 0.04 | 0.09 | 0.21 | 0.15 | 0.15 |
| *m*-Cymene | 1279 | 976 | 0.01 | | | | | | | | | | 0.01 | | | | | | | | |
| α-Terpinolene | 1291 | 1086 | 0.08 | | 0.11 | 0.04 | | 0.08 | 0.14 | 0.03 | | 0.12 | 0.05 | 0.10 | 0.06 | 0.09 | 0.06 | 0.09 | 0.05 | 0.08 | |
| Unknown I | | 1281 | 1.63 | **2.10** | 1.56 | | 1.65 | 1.56 | 1.42 | | **1.91** | 1.27 | 1.73 | 1.68 | 1.87 | | 1.84 | 1.72 | | 1.40 | **2.04** |
| 1,2,3,3-Tetramethyl-Cyclopenten-4-one | 1310 | | 0.24 | | 0.34 | | 0.30 | 0.46 | 0.32 | | 0.40 | 0.48 | 0.44 | 0.44 | 0.30 | 0.30 | 0.46 | 0.49 | | 0.44 | |
| Prenol | 1322 | 772 | | 0.01 | 0.02 | | 0.01 | 0.02 | 0.01 | | | | | 0.01 | | | | 0.01 | | 0.01 | |

**Table S2b. (**Continued).

| | | | 5 | | 6 | | | | | 7 | | | | | 8 | | | | | | |
|---|---|---|---|---|---|---|---|---|---|---|---|---|---|---|---|---|---|---|---|---|---|
| **Components** | **RI-WAX** | **RI-HP5** | **21** | **22** | **23** | **24** | **25** | **26** | **27** | **28** | **29** | **30** | **31** | **32** | **33** | **34** | **35** | **36** | **37** | **38** | **39** |
| *cis*-3-Hexenyl Acetate | 1328 | 1008 | 0.01 | | 0.02 | | 0.02 | 0.02 | 0.01 | | 0.02 | | 0.02 | 0.02 | | | | | | | |
| 6-Methyl-5-Hepten-2-one | 1351 | 986 | | | | 0.01 | 0.02 | 0.02 | 0.02 | 0.02 | 0.03 | 0.05 | | | | 0.02 | 0.01 | 0.02 | 0.01 | | 0.02 |
| *trans*-3-Hexenol | 1361 | 849 | 0.10 | | | | | | 0.16 | | | | 0.11 | | | 0.08 | 0.22 | | | | |
| n-Hexanol | 1365 | 861 | | 0.03 | | | 0.02 | | | | 0.03 | | | 0.04 | | 0.02 | 0.03 | 0.03 | | 0.03 | 0.04 |
| Alloocimene | 1384 | 1129 | | | 0.04 | 0.04 | | | | | | | | 0.04 | 0.06 | | | | 0.05 | | |
| 1-Octen-3-yl Acetate | 1387 | 1086 | | 0.04 | | | | | | | | | | 0.06 | | | | | | | |
| *cis*-3-Hexenol | 1390 | 853 | | 0.16 | 0.13 | 0.11 | 0.14 | 0.18 | | 0.16 | 0.19 | 0.12 | | 0.19 | 0.09 | 0.11 | 0.26 | 0.15 | 0.10 | 0.15 | 0.15 |
| n-Nonanal | 1406 | 1107 | | | | | | | | | 0.07 | | | | | | | | | | |
| *trans*-2-Hexenol | 1406 | 864 | 0.01 | | | | | | 0.01 | | | | | | | 0.01 | | | | | |
| Rosefuran | 1412 | 1104 | 0.03 | 0.03 | | 0.02 | | 0.02 | 0.02 | 0.02 | | | | | 0.04 | | 0.03 | 0.02 | 0.02 | 0.02 | |
| Fenchone | 1418 | 1090 | | | **3.94** | | **3.43** | | 0.34 | 1.10 | | | | | | **12.76** | 0.47 | | | | 0.17 |
| Unknown II | 1427 | 1083 | | | | 0.91 | | | | 0.84 | | | | | | | | | 0.78 | | |
| 1-Octen-3-ol | 1441 | 975 | | 0.14 | 0.09 | | | 0.16 | 0.17 | | 0.28 | 0.23 | | 0.22 | 0.06 | | | 0.36 | | 0.22 | 0.18 |
| Acetic Acid | 1453 | 505 | 0.01 | 0.24 | | | 0.19 | | | | 0.26 | | | 0.07 | | | 0.03 | 0.03 | | 0.02 | 0.25 |
| *cis*-Linalool Oxide (Furanoid) | 1460 | 1070 | 0.52 | 0.76 | 0.85 | 0.89 | 0.76 | 0.76 | 0.47 | 0.55 | 0.86 | 1.07 | 0.67 | 1.07 | 0.48 | 0.42 | 0.65 | 0.51 | 0.30 | 0.60 | 0.94 |
| Unknown III | 1471 | 1085 | 0.71 | 0.97 | 0.83 | | 0.76 | 1.02 | 0.75 | | 1.26 | 1.16 | 0.90 | 0.96 | 0.73 | 0.65 | 0.96 | 1.00 | | 0.81 | 1.04 |
| α-Cubebene | 1473 | 1351 | | | | 0.03 | 0.54 | | 0.05 | 0.06 | 0.05 | | | | | | | | 0.03 | | |
| *trans*-Linalool Oxide (Furanoid) | 1484 | 1086 | 0.42 | 0.57 | 0.62 | 0.73 | | 0.58 | 0.38 | 0.50 | 0.61 | 0.76 | 0.51 | 0.86 | 0.39 | 0.11 | 0.52 | 0.41 | 0.19 | 0.48 | 0.67 |
| Cyclosativene | 1493 | 1363 | 0.11 | 0.38 | 0.11 | 0.24 | 0.13 | 0.23 | 0.43 | 0.42 | 0.45 | 0.28 | 0.23 | 0.31 | 0.06 | 0.23 | 0.30 | 0.38 | 0.26 | 0.40 | 0.37 |
| α-Campholenal | 1509 | 1125 | 0.07 | | | | | | 0.13 | | | | | | | 0.06 | 0.05 | | | | |
| α-Copaene | 1511 | 1379 | 0.13 | 0.47 | 0.30 | 0.38 | 0.28 | 0.29 | 0.73 | 0.53 | 0.72 | 0.24 | 0.28 | 0.35 | 0.14 | 0.18 | 0.32 | 0.33 | 0.49 | 0.38 | 0.26 |
| Camphor | 1541 | 1149 | 0.82 | 0.82 | 1.73 | 1.01 | 1.57 | **2.98** | **6.27** | 1.03 | **2.71** | **7.61** | 0.92 | 1.08 | 0.64 | **2.72** | 0.38 | **2.15** | **6.42** | **2.90** | **2.54** |
| Cyperene | 1541 | 1390 | | | | | | | 0.13 | 0.14 | | | 0.07 | 0.07 | | | 0.08 | 0.07 | 0.09 | 0.08 | |
| α-Gurjunene | 1548 | 1407 | | | | | | | | | | | | | | | | | | | |
| *cis*-Sabinene Hydrate | 1550 | 1105 | | | | | | | | | | | | | | 0.04 | | | | | |

**Table S2b. (**Continued).

| | | | 5 | | 6 | | | | | 7 | | | | | 8 | | | | | | |
|---|---|---|---|---|---|---|---|---|---|---|---|---|---|---|---|---|---|---|---|---|---|
| **Components** | **RI-WAX** | **RI-HP5** | **21** | **22** | **23** | **24** | **25** | **26** | **27** | **28** | **29** | **30** | **31** | **32** | **33** | **34** | **35** | **36** | **37** | **38** | **39** |
| Unknown IV | | 1551 | 0.24 | 0.10 | 0.62 | 0.89 | 0.33 | 0.51 | 0.76 | 1.08 | 0.37 | 1.36 | 0.24 | 0.47 | 0.48 | 0.70 | 0.74 | 0.83 | | 0.52 | 0.21 |
| Unknown V | 1552 | | 0.24 | 0.40 | 0.30 | | 0.29 | 0.36 | 0.29 | | 0.49 | 0.33 | 0.31 | 0.34 | 0.29 | 0.20 | 0.33 | 0.33 | | 0.25 | 0.33 |
| Linalool | 1553 | 1100 | **3.15** | **4.63** | **4.97** | **4.67** | **5.63** | **4.07** | **3.56** | **3.72** | **5.61** | **4.62** | **4.04** | **6.58** | **2.96** | **2.10** | **3.30** | **2.86** | **2.39** | **2.91** | **4.55** |
| Linalyl Acetate | 1558 | 1255 | 0.08 | 0.12 | 0.17 | 0.10 | 0.24 | 0.13 | 0.08 | 0.09 | 0.17 | 0.18 | 0.08 | 0.12 | 0.05 | 0.07 | 0.09 | | 0.10 | 0.14 | 0.13 |
| Unknown VI | 1575 | 1156 | 0.51 | 0.41 | 0.57 | | 0.41 | 0.73 | 0.61 | | 0.31 | 0.57 | 0.73 | 0.66 | 0.67 | 0.43 | 0.59 | 0.69 | | 0.53 | 0.41 |
| Fenchol<*exo*> | 1582 | 1112 | | | | | | | | | | | | | | 0.24 | | | | | |
| Pinocarvone | 1589 | 1164 | 0.08 | 0.06 | 0.08 | | 0.08 | 0.13 | 0.09 | | 0.08 | 0.12 | | 0.07 | 0.07 | 0.10 | | | 0.03 | 0.09 | |
| γ-Amorphene | 1592 | 1490 | | | | | | | | | | | | | | | | | | 0.05 | |
| Bornyl Acetate | 1599 | 1288 | 0.07 | | 0.12 | | 0.14 | 0.13 | 0.08 | | | 0.15 | 0.07 | | | 0.21 | | | | 0.13 | 0.11 |
| *trans*-α-Necrodyl Acetate | 1606 | 1280 | **27.46** | **24.45** | **20.06** | **24.73** | **22.31** | **19.18** | **15.90** | **15.85** | **17.89** | **13.48** | **24.32** | **19.19** | **26.40** | **13.64** | **26.42** | **19.90** | **22.51** | **18.24** | **20.04** |
| Hotrienol | 1609 | 1108 | | | | | 0.10 | | | | | | | 0.09 | | | | | | | |
| Unknown VII | 1609 | 1326 | | | | 0.63 | | | | 0.60 | | | | | | | | | 0.64 | | |
| *trans*-β Caryophyllene | 1617 | 1450 | 0.24 | 0.35 | 0.38 | 0.49 | 0.30 | 0.41 | 0.88 | 0.93 | 0.31 | 0.41 | 0.42 | 0.48 | 0.22 | 0.36 | 0.49 | 0.59 | 0.72 | 0.63 | 0.35 |
| Lavandulyl Acetate | 1619 | 1284 | **3.65** | **6.96** | **5.34** | **6.03** | **5.76** | **5.16** | **4.39** | **5.26** | **6.14** | **3.73** | **4.78** | **5.85** | **5.85** | **3.55** | **4.93** | **5.50** | **4.57** | **4.02** | **6.35** |
| Terpinen-4-ol | 1622 | 1184 | 0.16 | | | 0.02 | 0.13 | | 0.05 | 0.06 | | | | | 0.05 | 0.19 | | | 0.06 | 0.12 | 0.08 |
| *cis*-α-Necrodyl Acetate | 1629 | 1302 | 1.81 | 1.27 | 1.52 | 1.61 | 1.62 | 1.08 | 0.91 | 1.20 | 1.01 | 0.84 | 1.67 | 1.42 | 1.37 | 1.16 | 1.80 | 1.13 | 1.48 | 1.04 | 1.30 |
| Arbozol | 1636 | 1296 | **1.86** | 1.58 | 1.82 | **1.89** | 1.53 | 2.15 | 0.91 | 1.37 | 1.02 | 1.57 | 1.85 | 2.01 | **2.55** | 1.57 | 1.96 | 1.87 | 1.57 | 1.58 | 1.75 |
| Valerena-4,7(11)-diene | 1637 | 1455 | | | | | | | | | | | | | | | | | | | |
| Sabina Ketone | 1645 | 1157 | | | | | | | | | | | | | | | | | 0.02 | | |
| Benzeneacetaldehyde | 1649 | 1049 | | | | | | | | 0.04 | 0.04 | | | | | | | | 0.04 | | 0.03 |
| Myrtenal | 1651 | 1197 | | | | 0.02 | | | | 0.05 | | 0.17 | | | | | | | 0.02 | | |
| Unknown VIII | 1654 | 1326 | 0.63 | 0.70 | 0.55 | | 0.60 | 0.53 | 0.41 | | 0.57 | 0.38 | 0.63 | 0.55 | 0.72 | 0.42 | 0.66 | 0.58 | | 0.46 | 0.62 |
| *cis*-Verbenol | 1660 | 1141 | | | | | | | | | | | | | | | | | | | |
| *trans*-Muurola-3,5-diene | 1661 | 1453 | 0.02 | | | | | | 0.04 | | | | 0.02 | | | 0.02 | 0.02 | | | | |
| Alloaromadendrene | 1664 | 1471 | 0.17 | 0.49 | 0.24 | 0.28 | | 0.25 | | 0.46 | 0.46 | | | 0.24 | 0.18 | 0.19 | 0.29 | 0.28 | 0.34 | 0.31 | 0.33 |

**Table S2b. (**Continued).

| | | | 5 | | 6 | | | | | 7 | | | | | 8 | | | | | | |
|---|---|---|---|---|---|---|---|---|---|---|---|---|---|---|---|---|---|---|---|---|---|
| **Components** | **RI-WAX** | **RI-HP5** | **21** | **22** | **23** | **24** | **25** | **26** | **27** | **28** | **29** | **30** | **31** | **32** | **33** | **34** | **35** | **36** | **37** | **38** | **39** |
| 5-Methylene-2,3,4,4-tetrame-2-Cyclopentenone | 1668 | 1187 | **2.18** | **2.56** | **2.46** | **2.82** | **2.20** | **3.14** | **2.95** | **2.92** | **3.16** | **3.04** | **3.78** | **2.85** | **2.08** | **2.23** | **3.29** | **3.83** | **3.03** | **4.13** | **3.32** |
| Lavandulol | 1669 | 1165 | 0.37 | 0.34 | 0.29 | 0.37 | 0.35 | 0.33 | 0.73 | 0.48 | 0.44 | 0.23 | 0.33 | 0.35 | 0.40 | 0.18 | 0.28 | 0.30 | 0.16 | | 0.34 |
| Unknown IX | 1673 | | 0.82 | 0.84 | 0.65 | | 0.70 | 0.44 | 0.62 | | 0.67 | 0.36 | 0.81 | 0.66 | 0.91 | 0.57 | 0.87 | 0.76 | | 0.56 | 0.76 |
| *trans*-α-Necrodol | 1679 | 1172 | **8.16** | **12.32** | **9.90** | **11.39** | **9.82** | **9.95** | **10.94** | **11.43** | **11.18** | **6.36** | **11.91** | **9.64** | **10.16** | **5.16** | **9.08** | **10.27** | **6.89** | **7.95** | **8.20** |
| *trans*-Cadina-1(6),4-diene | 1682 | 1477 | 0.07 | | 0.09 | 0.10 | | 0.11 | 0.11 | 0.12 | | 0.06 | 0.10 | 0.11 | 0.06 | 0.08 | 0.09 | 0.10 | 0.10 | 0.11 | |
| *trans*-Verbenol | 1692 | 1151 | 0.27 | 0.49 | 0.20 | | 0.22 | 0.38 | 0.21 | | 0.36 | 0.99 | 0.39 | 0.45 | 0.24 | 0.46 | 0.28 | 0.49 | | 0.15 | 0.16 |
| α-Humulene | 1694 | 1454 | | | | | | | | 0.15 | | | | | 0.17 | | | | 0.11 | | |
| β-Acoradiene | 1694 | 1466 | | | | | | | | | | | | | | | | | | | |
| α-Terpineol | 1696 | 1195 | 0.32 | | 0.09 | 0.05 | 0.18 | 0.10 | 0.06 | 0.02 | 0.07 | 0.12 | 0.05 | | | 0.11 | 0.05 | | 0.07 | 0.13 | 0.16 |
| γ-Muurolene | 1700 | 1485 | 0.02 | | | 0.05 | 0.07 | 0.04 | 0.07 | 0.09 | | | 0.04 | | 0.03 | 0.03 | 0.05 | 0.05 | 0.06 | 0.05 | 0.06 |
| Heptadecane | 1700 | 1700 | 0.02 | | | 0.04 | | | 0.03 | 0.06 | | | 0.05 | | | 0.05 | 0.06 | | | | |
| Myrtenyl Acetate | 1702 | 1329 | 0.30 | | | | | | 0.02 | | | 0.65 | 0.09 | 0.10 | | 0.26 | 0.17 | 0.18 | 0.14 | 0.15 | 0.23 |
| Ledene | 1708 | 1485 | 0.03 | | | | | 0.05 | 0.07 | | 0.05 | 0.06 | 0.05 | 0.06 | 0.02 | | 0.06 | 0.06 | | 0.06 | |
| α-Amorphene | 1711 | 1506 | | | | | | | | | | | | | | | | | | | |
| Borneol | 1713 | 1167 | | | | | | | | | | | | | | | | | 0.07 | | |
| Neryl Acetate | 1723 | 1361 | 0.41 | 0.66 | 0.54 | 0.85 | 0.44 | 0.25 | 0.60 | 0.52 | 0.80 | 0.16 | 0.66 | 0.35 | 0.79 | 0.19 | 0.87 | 0.71 | 0.60 | 0.44 | 0.66 |
| 10-Epizonarene | 1725 | 1506 | | | | 0.06 | | | | | | | | | | | | | | | |
| *cis*-Muurola-4(14),5-diene | 1735 | 1466 | | | | | | | 0.42 | | | | 0.29 | 0.13 | | | | | | | |
| *cis*-α-Necrodol | 1735 | 1150 | 1.51 | **2.16** | 1.76 | **1.95** | 1.58 | 1.85 | 1.84 | **1.95** | **1.96** | 1.10 | 2.09 | **2.02** | **2.04** | 1.46 | **2.06** | 2.00 | **1.60** | 1.37 | **1.87** |
| Eremophilene | 1735 | 1486 | | | | | | | | | | | | | | | | | | | |
| Valencene | 1736 | 1409 | | | | | | | | | | 0.04 | | | | | | | | | |
| Verbenone | 1736 | 1208 | 0.16 | 0.28 | | 0.22 | | 0.33 | 0.20 | 0.22 | 0.19 | 0.47 | 0.15 | | 0.13 | 0.22 | 0.09 | 0.17 | 0.12 | | 0.21 |
| α-Selinene | 1737 | 1501 | 0.09 | 0.19 | 0.17 | | 0.20 | 0.17 | 0.22 | | 0.26 | 0.27 | 0.10 | 0.22 | 0.11 | 0.16 | 0.19 | 0.15 | | 0.15 | 0.15 |
| Unknown X | 1739 | 1173 | | | | **2.02** | | | | **2.08** | | | | | | | | | 1.24 | | |
| α-Muurolene | 1746 | 1501 | | 0.13 | | 0.20 | | | | 0.44 | | | | | | | | | 0.33 | | |

**Table S2b. (**Continued).

| | | | 5 | | 6 | | | | | 7 | | | | | 8 | | | | | | |
|---|---|---|---|---|---|---|---|---|---|---|---|---|---|---|---|---|---|---|---|---|---|
| **Components** | **RI-WAX** | **RI-HP5** | **21** | **22** | **23** | **24** | **25** | **26** | **27** | **28** | **29** | **30** | **31** | **32** | **33** | **34** | **35** | **36** | **37** | **38** | **39** |
| β-Selinene | 1746 | 1502 | 0.16 | 0.25 | 0.49 | 0.45 | 0.32 | 0.28 | 0.41 | 0.44 | 0.69 | 0.49 | 0.13 | 0.32 | 0.26 | 0.37 | 0.40 | 0.44 | 0.35 | 0.28 | 0.33 |
| Geranyl Acetate | 1758 | 1380 | 0.27 | 0.10 | 0.18 | 0.32 | 0.25 | 0.13 | 0.07 | 0.10 | 0.07 | 0.10 | 0.08 | 0.09 | 0.19 | 0.41 | 0.13 | 0.15 | 0.44 | 0.36 | 0.28 |
| (E,E) α Famesene | 1760 | 1508 | | | | | | | | 0.24 | | | | | | | | | | | |
| Carvone | 1762 | 1246 | 0.02 | | | 0.01 | | | 0.04 | 0.03 | | | 0.02 | | | 0.10 | 0.02 | | 0.02 | | |
| *trans*-Linalool Oxide (Pyranoid) | 1767 | 1170 | 0.03 | | | | | | 0.02 | | | 0.05 | 0.02 | 0.05 | | 0.03 | 0.03 | | | | |
| δ-Cadinene | 1776 | 1524 | 0.56 | 0.62 | 0.97 | 0.91 | 0.67 | 1.15 | 1.38 | 1.45 | 0.35 | 0.98 | 1.04 | 1.29 | 0.54 | 0.60 | 1.01 | 0.94 | 1.24 | 1.07 | 0.62 |
| Unknown XI | 1793 | 1173 | **1.94** | 1.39 | **1.83** | | 1.39 | 1.97 | **2.27** | | 1.06 | 1.36 | **2.11** | 1.87 | **2.02** | | 1.69 | 1.89 | | 1.37 | 1.40 |
| γ-Cadinene | 1782 | 1515 | 0.29 | 0.72 | 0.42 | 0.41 | 0.47 | 0.44 | 0.88 | 0.88 | 0.84 | | 0.51 | 0.52 | | 0.26 | 0.57 | 0.60 | | 0.54 | 0.62 |
| 1,7,7-Trimethylbicyclo[2.2.1]hept-5-en-2-ol | 1785 | 1120 | | | 0.08 | | | | | 0.07 | | | | 0.07 | | | | | | | |
| Myrtenol | 1798 | 1202 | | | | | | | | | | | | | | | | | 0.56 | | |
| *p*-Methyl Acetophenone | 1800 | 1183 | | | | | | | | | | | | | | | | | | | |
| Eudesma-3,7(11)-diene | 1805 | 1532 | 0.49 | 0.25 | 1.65 | 1.77 | 0.89 | 0.95 | 1.62 | **2.21** | 1.16 | **3.24** | 0.49 | 1.01 | 0.92 | **1.76** | 1.47 | 1.61 | 1.00 | 0.99 | 0.54 |
| α-Cadinene | 1817 | 1538 | | | | | | | | | | | | | | | | | | | |
| *trans*-Carveol | 1822 | 1223 | | | | | | | | | | | | | | | | | | | |
| Phenethyl Acetate | 1828 | 1257 | | 0.11 | 0.24 | 0.29 | 0.27 | 0.10 | | | | | | 0.17 | | | | | 0.22 | | |
| *p*-Cymen-8-ol | 1839 | 1189 | 0.03 | 0.08 | 0.08 | 0.04 | 0.04 | 0.10 | 0.12 | 0.05 | 0.11 | | 0.04 | | | 0.03 | 0.03 | | | | 0.11 |
| *cis*-Carveol | 1852 | 1232 | 0.05 | 0.05 | | | 0.06 | 0.08 | 0.06 | 0.05 | | 0.09 | 0.03 | | | 0.10 | | | | | 0.04 |
| Unknown XII | 1852 | 1393 | | | | | | | | | | | | | | | | | | | |
| Germacrene B | 1853 | 1557 | | | | | | | | | | | | | | | | | | | |
| *trans*-Calamenene | 1857 | 1527 | 0.09 | 0.22 | | | | | 0.17 | | 0.24 | | 0.11 | | | | 0.08 | | | | |
| Unknown XIII | | 1860 | **2.14** | **2.24** | **2.26** | | **2.13** | **2.82** | **2.70** | | 1.57 | 1.83 | **3.01** | **2.97** | 1.86 | 1.73 | **2.92** | **3.21** | | **3.00** | 1.83 |
| Unknown XIV | | 1861 | | | | 0.91 | | | | 1.08 | | | | | | | | | 0.93 | | |
| *meta*-Cymen-8-ol | 1861 | 1183 | 0.25 | | | | | | 0.14 | | | | 0.30 | | | 0.18 | 0.25 | | | | |
| Unknown XV | 1862 | 1393 | 0.89 | | | | | | 0.45 | | | | 1.00 | | | 0.79 | 0.82 | | | | |
| Unknown XVI | | 1870 | 1.68 | **1.69** | 1.80 | 0.70 | **1.68** | 2.02 | 2.02 | 0.81 | 1.30 | 1.23 | **2.32** | **2.24** | 1.53 | 1.40 | **2.30** | **2.50** | 0.72 | **2.29** | 1.45 |

**Table S2b. (**Continued).

| | | | 5 | | 6 | | | | | 7 | | | | | 8 | | | | | | |
|---|---|---|---|---|---|---|---|---|---|---|---|---|---|---|---|---|---|---|---|---|---|
| **Components** | **RI-WAX** | **RI-HP5** | **21** | **22** | **23** | **24** | **25** | **26** | **27** | **28** | **29** | **30** | **31** | **32** | **33** | **34** | **35** | **36** | **37** | **38** | **39** |
| Cubebol Isomer I | 1903 | 1516 | 0.46 | | | 0.19 | | | | | | | 0.73 | | | | | | 0.56 | | |
| *epi*-Cubebol | 1903 | 1498 | 0.36 | 0.81 | 0.42 | 0.89 | 0.76 | 0.49 | 0.94 | 1.49 | 1.20 | 0.40 | 0.61 | 0.56 | 0.30 | 0.28 | 0.64 | 0.65 | 0.52 | 0.90 | 0.68 |
| 10,11-Epoxycalamenene | 1903 | 1498 | | | | | | | | | | | | | | | | | | | |
| *cis*-Muurol-5-en-4-β-ol | 1905 | 1548 | | | | | | | | 0.71 | | | | | | | | | | | |
| Unknown XVII | 1946 | 1564 | | | | 0.81 | | | | 0.98 | | | | | | | | | 0.76 | | |
| α-Calacorene | 1944 | 1542 | 0.09 | 0.12 | 0.10 | | | 0.14 | 0.16 | | | 0.16 | 0.14 | 0.13 | 0.09 | 0.09 | 0.13 | 0.17 | | 0.14 | 0.09 |
| Palustrol | 1958 | 1568 | 0.05 | 0.08 | | 0.06 | | 0.08 | 0.08 | 0.10 | 0.10 | | 0.07 | | | 0.04 | 0.08 | | 0.08 | 0.08 | |
| *trans*-Muurol-5-en-4-β-ol | 1958 | | | 0.61 | 0.38 | | 0.48 | 0.41 | 1.02 | | 0.68 | 0.29 | 0.65 | 0.48 | 0.22 | 0.27 | 0.66 | 0.48 | | 0.61 | 0.54 |
| n-Dodecanol | 1975 | 1476 | | | | | | | | | | | | | | | | | | | |
| Unknown XVIII | 1996 | 1564 | 0.68 | 1.09 | 0.59 | | 0.80 | 0.66 | 1.09 | | 1.00 | 0.54 | 0.76 | 0.89 | 0.54 | 0.47 | 0.71 | 0.79 | | 0.60 | 0.76 |
| Caryophyllene Oxide | 2014 | 1578 | 0.32 | 0.88 | 0.35 | 0.43 | 0.65 | 0.51 | 0.55 | 0.61 | 1.22 | 0.51 | 0.42 | 0.73 | 0.29 | 0.30 | 0.45 | 0.54 | 0.37 | 0.46 | 0.74 |
| Unknown XIX | 2059 | 1578 | | | | 1.09 | | | | 1.50 | | | | | | | | | 1.28 | | |
| Ledol | 2066 | 1574 | 0.42 | 0.75 | 0.47 | 0.47 | 0.56 | 0.68 | 0.69 | 0.65 | 0.97 | 0.79 | 0.58 | 0.67 | 0.34 | 0.38 | 0.47 | 0.71 | 0.57 | 0.69 | 0.70 |
| 1,10-Di-*epi*-Cubenol | 2099 | 1614 | 0.32 | 0.30 | | | 0.16 | | 0.49 | | | | 0.43 | | 0.24 | | 0.15 | 0.20 | | 0.17 | |
| Viridiflorol | 2103 | 1610 | 1.45 | **2.64** | 1.76 | **2.29** | **2.27** | **2.89** | **2.57** | **3.11** | **3.43** | **2.96** | **2.29** | **2.46** | 1.24 | 1.44 | **2.37** | **2.71** | **3.09** | **2.73** | **2.74** |
| 1-*epi*-Cubenol | 2107 | 1613 | | 0.52 | 0.31 | 0.37 | 0.39 | 0.44 | | 0.57 | 0.52 | 0.44 | | 0.44 | 0.29 | 0.27 | 0.41 | 0.47 | 0.50 | 0.47 | 0.46 |
| Unknown XX | 2115 | 1577 | 0.92 | 1.68 | 0.84 | | 1.16 | 0.80 | 2.09 | | 1.38 | 0.58 | 0.72 | 1.26 | 0.67 | 0.60 | 1.05 | 1.05 | | 1.05 | 0.89 |
| Spathulenol | 2149 | 1572 | | | | | | | | 0.09 | 0.14 | | | | | | | | | | |
| Copaborneol | 2199 | 1613 | 0.77 | 1.38 | 0.86 | 1.08 | 1.03 | 1.28 | 1.35 | 1.50 | **1.78** | 1.48 | 1.18 | 1.32 | 0.59 | 0.72 | 1.20 | 1.40 | 1.25 | 1.43 | 1.38 |
| (E)-1-(6,10-Dimethylundec-5-en-2-yl)-4-Methylbenzene | 2212 | | | | | 0.20 | | | | 0.24 | | | | | | | 0.13 | | 0.27 | | |
| Pentylcurcumene | 2212 | | | | 0.24 | | | 0.18 | | | | | | 0.28 | | 0.14 | | | | | |
| T-Muurolol | 2215 | 1645 | 0.12 | 0.38 | 0.31 | 0.27 | 0.24 | 0.41 | 0.38 | 0.45 | 0.37 | 0.25 | 0.17 | 0.25 | 0.28 | 0.16 | 0.44 | 0.26 | 0.36 | 0.24 | 0.20 |
| α-Muurolol | 2224 | 1649 | 0.07 | | | | | | 0.11 | 0.13 | | | 0.05 | | | 0.11 | | | 0.14 | | |
| Cadalene | 2241 | 1670 | 0.06 | 0.15 | 0.08 | 0.07 | 0.11 | 0.12 | 0.10 | 0.09 | 0.19 | 0.14 | 0.08 | 0.10 | 0.05 | 0.07 | 0.09 | 0.11 | 0.09 | 0.10 | 0.13 |
| α-Cadinol | 2249 | 1652 | 0.02 | | 1.03 | | 0.50 | 0.27 | 0.03 | | | | | | | 0.31 | 0.16 | 0.19 | | | |

**Table S2b. (**Continued).

| | | | 5 | | 6 | | | | | 7 | | | | | 8 | | | | | | |
|---|---|---|---|---|---|---|---|---|---|---|---|---|---|---|---|---|---|---|---|---|---|
| **Components** | **RI-WAX** | **RI-HP5** | **21** | **22** | **23** | **24** | **25** | **26** | **27** | **28** | **29** | **30** | **31** | **32** | **33** | **34** | **35** | **36** | **37** | **38** | **39** |
| β-Eudesmol | 2251 | 1656 | | | | 0.09 | | | | 0.09 | | | | | | | | | | | |
| *neo*-Intermedeol | 2264 | 1661 | | | | | | | | 0.04 | | | | | | | | | | | |
| (E)-1-(6,10-Dimethylundec-5,9-dien-2-yl)-4-Methylbenzene | 2293 | | | | | | | | | | | | | | | | | | | | |
| Pentenylcurcumene | 2293 | 1970 | | | | | 0.09 | | | | | | | | | | | | | | 0.12 |
| Eudesm-7(11)-en-4-ol | 2312 | 1696 | 0.12 | | 0.24 | 0.32 | 0.16 | 0.22 | 0.23 | 0.34 | 0.19 | 0.63 | 0.08 | 0.18 | 0.19 | 0.22 | 0.23 | 0.33 | 0.27 | | |
| *cis*-14-nor-Muurol-5-en-4-one | 2329 | 1696 | 0.04 | | 0.15 | | 0.14 | | 0.10 | | | | 0.05 | | | 0.03 | 0.01 | | | | |
| Vetiveryl Acetate Isomer | 2342 | 1872 | | | | | | | | | | 0.22 | | | | | | | | | |
| α-Cyperone | 2370 | 1706 | | | | 0.09 | | | | 0.07 | | | | | | | | | 0.06 | | |
| Phenethyl Phenylacetate | 2817 | 1915 | | | | 0.10 | 0.08 | 0.05 | | | | | | | | | | | | | |
| **% Identification** | | | 91.75 | 90.72 | 90.26 | 85.70 | 91.16 | 89.53 | 91.74 | 81.79 | 86.57 | 83.70 | 90.88 | 88.79 | 88.04 | 86.40 | 91.16 | 88.70 | 84.19 | 91.21 | 87.62 |
| **Yield (g/kg)** | | | 4.30 | 2.32 | 2.95 | 2.22 | 2.63 | 1.91 | 1.42 | 1.09 | 1.08 | 1.26 | 1.83 | 2.05 | 5.93 | 4.31 | 1.97 | 1.48 | 1.55 | 1.34 | 1.74 |
| Monoterpene hydrocarbons | | | 6.62 | 2.99 | 6.11 | 4.58 | 3.84 | 6.11 | 5.24 | 4.39 | 1.18 | 8.27 | 4.40 | 5.11 | 6.01 | 5.67 | 4.09 | 4.78 | 4.67 | 4.85 | 2.67 |
| Oxygen-containing monoterpenes | | | 60.39 | 56.45 | 55.06 | 55.44 | 59.22 | 51.04 | 50.02 | 44.95 | 51.51 | 44.38 | 53.47 | 50.21 | 57.63 | 59.15 | 52.08 | 47.79 | 54.72 | 53.66 | 55.89 |
| Sesquiterpene hydrocarbons | | | 2.62 | 4.34 | 5.00 | 5.44 | 3.98 | 4.63 | 7.87 | 8.65 | 5.77 | 6.37 | 4.10 | 5.34 | 2.85 | 4.40 | 5.64 | 5.88 | 5.21 | 5.34 | 3.85 |
| Oxygen-containing sesquiterpenes | | | 4.48 | 8.35 | 6.13 | 6.55 | 7.20 | 7.68 | 8.44 | 9.95 | 10.60 | 7.97 | 7.26 | 7.09 | 3.98 | 4.50 | 7.26 | 7.94 | 7.77 | 7.78 | 7.44 |
| Unknown | | | 4.61 | 4.98 | 5.56 | 5.73 | 5.02 | 6.65 | 4.69 | 4.88 | 5.62 | 5.74 | 6.38 | 6.49 | 5.28 | 4.72 | 6.61 | 6.96 | 5.47 | 6.74 | 6.03 |
| Other | | | 13.03 | 13.61 | 12.40 | 7.96 | 11.90 | 13.42 | 15.48 | 8.97 | 11.89 | 10.97 | 15.27 | 14.55 | 12.29 | 7.96 | 15.48 | 15.35 | 6.35 | 12.84 | 11.74 |

**Table S2c.** Chemical composition of *L. stoechas* subsp. *luisieri* (Zone: 9. Serra de São Mamede, 10. Sierra de San Pedro, 11. Sierra de Aracena y Picos de Aroche).

| | | | **9** | | **10** | | | | | **11** | | | | | | | | |
|---|---|---|---|---|---|---|---|---|---|---|---|---|---|---|---|---|---|---|
| **Components** | **RI-WAX** | **RI-HP5** | **40** | **41** | **42** | **43** | **44** | **45** | **46** | **47** | **48** | **49** | **50** | **51** | **52** | **53** | **54** | **55** |
| α-Pinene | 1025 | 933 | 1.12 | 1.79 | **1.93** | 1.81 | 1.75 | 1.56 | 1.95 | **1.83** | 0.96 | **2.12** | **1.96** | 0.84 | 0.89 | 0.89 | 1.75 | 1.31 |
| Methyl Butenol | 1043 | | 0.28 | 0.14 | 0.11 | 0.14 | 0.14 | 0.40 | 0.10 | 0.12 | 0.08 | 0.09 | 0.13 | 0.19 | 0.07 | 0.10 | 0.07 | 0.11 |
| α-Fenchene | 1060 | 948 | | 0.01 | 0.01 | | <0,01 | | | | | | | | | | | |
| Camphene | 1069 | 953 | 0.21 | 0.11 | 0.27 | 0.10 | 0.10 | 0.12 | 0.06 | 0.10 | 0.09 | | 0.08 | 0.05 | 0.07 | 0.07 | 0.09 | 0.07 |
| n-Hexanal | 1090 | 796 | | | | 0.03 | 0.02 | 0.02 | | | 0.04 | 0.05 | 0.02 | 0.03 | | | 0.02 | 0.04 |
| β-Pinene | 1114 | 978 | 0.04 | 0.05 | 0.03 | 0.12 | 0.02 | 0.04 | 0.02 | 0.30 | 0.13 | 0.28 | 0.04 | 0.11 | 0.23 | 0.02 | 0.07 | 0.15 |
| Sabinene | 1126 | 972 | | 0.02 | | 0.06 | | | | 0.12 | 0.07 | 0.13 | | 0.05 | 0.08 | | 0.05 | 0.06 |
| Verbenene | 1130 | 971 | 0.04 | 0.13 | 0.07 | 0.05 | 0.09 | 0.08 | 0.06 | 0.06 | 0.03 | 0.06 | 0.04 | 0.04 | 0.03 | 0.04 | 0.07 | 0.05 |
| Cymene Isomer | 1133 | 940 | **2.46** | **3.27** | **2.28** | 0.26 | **2.82** | **3.29** | **2.34** | **2.67** | **2.46** | 1.83 | 0.57 | **2.33** | **2.29** | **2.18** | **2.02** | **2.68** |
| p-Xylene | 1146 | 865 | 0.01 | 0.01 | <0,01 | | <0,01 | 0.01 | <0.01 | <0,01 | | | | 0.01 | 0.01 | <0,01 | | |
| δ-3-Carene | 1158 | 1009 | 0.41 | 0.12 | 0.20 | 0.11 | 0.37 | 0.21 | 0.29 | 0.03 | 0.03 | 0.03 | 0.06 | 0.09 | 0.04 | 0.06 | 0.04 | 0.03 |
| β-Myrcene | 1165 | 991 | 0.04 | 0.05 | 0.07 | 0.07 | 0.05 | 0.05 | 0.04 | 0.07 | 0.04 | 0.08 | 0.05 | 0.05 | 0.06 | 0.04 | 0.05 | 0.07 |
| 3-Methyl-2-Butenal | 1194 | 779 | 0.04 | 0.06 | 0.04 | 0.04 | 0.04 | 0.05 | 0.04 | 0.04 | 0.04 | 0.04 | 0.04 | 0.03 | 0.03 | 0.03 | 0.03 | 0.04 |
| Limonene | 1206 | 1021 | 0.25 | 0.21 | 0.22 | 0.21 | 0.17 | 0.14 | 0.19 | 0.20 | 0.14 | 0.21 | 0.21 | 0.13 | 0.17 | 0.15 | 0.16 | 0.14 |
| 1,8-Cineole | 1222 | 1039 | 0.04 | 1.19 | 0.49 | **5.35** | 0.49 | 1.68 | **2.16** | **17.71** | **6.35** | **10.69** | 1.35 | **5.63** | **12.84** | 0.46 | **6.43** | **8.47** |
| *cis*-β-Ocimene | 1238 | 1035 | 1.02 | 0.58 | 0.64 | 0.19 | 1.20 | 1.19 | 0.99 | 0.45 | 0.71 | 0.82 | 0.37 | 0.71 | 0.45 | 0.99 | 0.13 | 0.65 |
| γ-Terpinene | 1254 | 1058 | 0.01 | | | | | 0.01 | | 0.12 | | | | 0.07 | 0.11 | | | |
| *trans*-β Ocimene | 1256 | 1046 | 0.06 | | 0.05 | | 0.08 | 0.07 | 0.07 | 0.04 | | 0.05 | | 0.17 | 0.06 | 0.06 | 0.16 | 0.11 |
| Prenyl Acetate | 1259 | 920 | 0.03 | 0.06 | 0.04 | 0.05 | 0.05 | 0.03 | 0.05 | 0.04 | 0.05 | 0.04 | 0.05 | 0.02 | 0.04 | 0.04 | 0.04 | 0.04 |
| *p*-Cymene | 1278 | 1025 | 0.07 | 0.10 | 0.10 | 0.16 | 0.13 | 0.07 | 0.11 | 0.14 | 0.07 | 0.13 | 0.11 | 0.05 | 0.09 | 0.07 | 0.13 | 0.11 |
| *m*-Cymene | 1279 | 976 | 0.03 | 0.02 | 0.02 | 0.01 | 0.03 | 0.02 | 0.03 | | | | | 0.01 | | | | |
| α-Terpinolene | 1291 | 1086 | 0.09 | | 0.05 | | 0.08 | 0.03 | 0.05 | 0.04 | 0.05 | 0.05 | | 0.06 | 0.07 | 0.05 | 0.04 | 0.07 |
| Unknown I | | 1281 | | 1.58 | 1.62 | **1.95** | 1.72 | | 1.60 | 1.47 | 1.65 | 1.73 | **2.06** | | 1.60 | 1.64 | 1.64 | 1.67 |
| 1,2,3,3-Tetramethyl-Cyclopenten-4-one | 1310 | | | 0.51 | 0.37 | 0.32 | 0.41 | | 0.38 | 0.27 | 0.37 | 0.24 | 0.30 | | 0.35 | 0.40 | | 0.44 |
| Prenol | 1322 | 772 | | | | 0.02 | | | | | 0.01 | 0.01 | 0.01 | 0.01 | | | | 0.01 |

**Table S2c.** (Continued).

| | | | 9 | | 10 | | | | | 11 | | | | | | | | |
|---|---|---|---|---|---|---|---|---|---|---|---|---|---|---|---|---|---|---|
| **Components** | **RI-WAX** | **RI-HP5** | **40** | **41** | **42** | **43** | **44** | **45** | **46** | **47** | **48** | **49** | **50** | **51** | **52** | **53** | **54** | **55** |
| *cis*-3-Hexenyl Acetate | 1328 | 1008 | 0.01 | 0.01 | 0.01 | | 0.02 | 0.01 | 0.02 | 0.01 | | 0.01 | | | 0.01 | 0.01 | | |
| 6-Methyl-5-Hepten-2-one | 1351 | 986 | 0.01 | 0.02 | 0.02 | 0.02 | 0.02 | 0.01 | 0.01 | 0.01 | 0.01 | 0.01 | | 0.01 | 0.01 | 0.02 | | |
| *trans*-3-Hexenol | 1361 | 849 | 0.09 | 0.19 | 0.08 | | 0.11 | | 0.14 | 0.11 | | | | | | | 0.06 | |
| n-Hexanol | 1365 | 861 | | | | | | | 0.03 | 0.01 | | | | | 0.01 | 0.02 | 0.02 | |
| Alloocimene | 1384 | 1129 | 0.06 | | | | | 0.05 | | | | | | 0.03 | | 0.04 | | |
| 1-Octen-3-yl Acetate | 1387 | 1086 | | | | | | | | | | | | | | | 0.04 | |
| *cis*-3-Hexenol | 1390 | 853 | 0.09 | | | 0.11 | | 0.13 | | 0.13 | 0.09 | 0.08 | 0.09 | 0.11 | 0.05 | 0.12 | 0.09 | 0.07 |
| n-Nonanal | 1406 | 1107 | | | 0.05 | | 0.06 | | 0.04 | 0.04 | | | | | 0.06 | 0.05 | | |
| *trans*-2-Hexenol | 1406 | 864 | | 0.04 | 0.01 | 0.07 | 0.08 | 0.04 | 0.06 | 0.02 | 0.02 | 0.02 | 0.02 | | 0.02 | 0.05 | 0.04 | |
| Rosefuran | 1412 | 1104 | 0.03 | | 0.02 | 0.02 | 0.03 | 0.02 | 0.02 | | 0.02 | 0.02 | 0.03 | 0.01 | 0.02 | 0.03 | | 0.02 |
| Fenchone | 1418 | 1090 | | 1.26 | 0.88 | 1.04 | 0.15 | 0.71 | | 0.28 | 0.29 | 0.01 | 0.39 | 0.03 | 0.45 | | | |
| Unknown II | 1427 | 1083 | 0.86 | 1.32 | | | | 0.96 | | | | | | 0.86 | | | | |
| 1-Octen-3-ol | 1441 | 975 | | | | | | | | | | | | | | | 0.27 | |
| Acetic Acid | 1453 | 505 | | | | 0.25 | | | | | 0.04 | 0.10 | 0.28 | | | | 0.07 | |
| *cis*-Linalool Oxide (Furanoid) | 1460 | 1070 | 0.74 | 1.27 | 0.90 | 1.07 | 0.60 | 0.83 | 0.76 | 0.41 | 0.44 | 0.44 | 0.67 | 0.60 | 0.42 | 0.47 | 0.83 | 0.65 |
| Unknown III | 1471 | 1085 | | | 0.92 | 1.10 | 1.01 | | 1.03 | 0.73 | 0.88 | 0.73 | 0.27 | | 0.80 | 0.87 | 0.89 | 0.86 |
| $\alpha$-Cubebene | 1473 | 1351 | 0.03 | | | | | 0.02 | | | | | | 0.03 | | | | |
| *trans*-Linalool Oxide (Furanoid) | 1484 | 1086 | 0.63 | 0.95 | 0.70 | 0.80 | 0.43 | 0.70 | 0.58 | 0.33 | 0.35 | 0.35 | 0.50 | 0.53 | 0.33 | 0.36 | 0.66 | 0.53 |
| Cyclosativene | 1493 | 1363 | 0.32 | 0.27 | 0.19 | 0.16 | 0.13 | 0.18 | 0.14 | 0.15 | 0.25 | 0.22 | 0.27 | 0.28 | 0.19 | 0.41 | 0.33 | 0.20 |
| $\alpha$-Campholenal | 1509 | 1125 | | | 0.04 | | 0.11 | | 0.05 | 0.05 | | | | | 0.03 | | 0.08 | |
| $\alpha$-Copaene | 1511 | 1379 | 0.47 | 0.26 | 0.18 | 0.11 | 0.07 | 0.13 | 0.15 | 0.15 | 0.24 | 0.16 | 0.24 | 0.38 | 0.17 | 0.43 | 0.25 | 0.32 |
| Camphor | 1541 | 1149 | **4.90** | **2.08** | **2.45** | **1.90** | 1.94 | **2.04** | 0.98 | 1.00 | 1.52 | 0.70 | 1.20 | 0.40 | 0.92 | 1.18 | 1.53 | 0.95 |
| Cyperene | 1541 | 1390 | 0.07 | 0.06 | 0.04 | | 0.06 | 0.03 | 0.05 | 0.05 | | | | 0.07 | | | 0.05 | |
| $\alpha$-Gurjunene | 1548 | 1407 | | | 0.06 | | | | 0.05 | | | | | | | | | |
| *cis*-Sabinene Hydrate | 1550 | 1105 | | | | | | | | 0.06 | | | | | 0.05 | | | |

**Table S2c.** (Continued).

| | | | 9 | | 10 | | | | | 11 | | | | | | | | |
|---|---|---|---|---|---|---|---|---|---|---|---|---|---|---|---|---|---|---|
| **Components** | **RI-WAX** | **RI-HP5** | **40** | **41** | **42** | **43** | **44** | **45** | **46** | **47** | **48** | **49** | **50** | **51** | **52** | **53** | **54** | **55** |
| Unknown IV | | 1551 | | 0.42 | 0.90 | 0.25 | 0.25 | | 0.40 | 0.33 | 0.45 | 0.19 | | | 0.32 | 0.32 | 0.64 | 0.29 |
| Unknown V | 1552 | | | 0.56 | 0.43 | 0.37 | 0.39 | | 0.42 | 0.30 | | 0.21 | 0.84 | | 0.35 | 0.35 | 0.28 | 0.33 |
| Linalool | 1553 | 1100 | **3.98** | **4.75** | **4.84** | **5.25** | **4.00** | **4.89** | **3.99** | **2.29** | **3.00** | **2.91** | **4.23** | **3.79** | **2.46** | **2.54** | **4.99** | **3.77** |
| Linalyl Acetate | 1558 | 1255 | 0.09 | 0.07 | 0.08 | 0.10 | 0.04 | 0.06 | 0.06 | 0.08 | 0.09 | 0.10 | 0.12 | 0.13 | 0.05 | 0.07 | 0.11 | 0.11 |
| Unknown VI | 1575 | 1156 | | 0.78 | 0.41 | 0.34 | 0.56 | | 0.44 | 0.46 | 0.60 | 0.42 | 0.38 | | 0.39 | 0.47 | 0.59 | 0.63 |
| Fenchol<*exo*> | 1582 | 1112 | | | | | | | | | | | | | | | | |
| Pinocarvone | 1589 | 1164 | 0.05 | | | | | 0.05 | | 0.13 | | 0.10 | | | 0.12 | | 0.10 | 0.09 |
| γ-Amorphene | 1592 | 1490 | 0.05 | | | | | 0.03 | | | | | | 0.05 | | | | |
| Bornyl Acetate | 1599 | 1288 | 0.05 | 0.09 | 0.24 | 0.10 | 0.07 | 0.05 | 0.17 | 0.06 | 0.06 | 0.07 | 0.14 | 0.05 | 0.03 | 0.05 | 0.09 | |
| *trans*-α-Necrodyl Acetate | 1606 | 1280 | **19.08** | **17.58** | **24.97** | **26.83** | **23.09** | **21.22** | **25.15** | **20.46** | **24.00** | **28.15** | **30.50** | **21.00** | **24.42** | **22.57** | **17.54** | **20.54** |
| Hotrienol | 1609 | 1108 | | | 0.10 | | | | 0.09 | 0.11 | | | | | | | | |
| Unknown VII | 1609 | 1326 | 0.54 | | | | | 0.70 | | | | | | 0.73 | | | | |
| *trans*-β Caryophyllene | 1617 | 1450 | 0.44 | 0.27 | 0.42 | 0.16 | 0.28 | 0.28 | 0.44 | 0.22 | 0.27 | 0.23 | 0.22 | 0.46 | 0.38 | 0.65 | 0.37 | 0.33 |
| Lavandulyl Acetate | 1619 | 1284 | **5.37** | **3.72** | **4.24** | **4.65** | **4.39** | **5.80** | **4.02** | **4.01** | **5.24** | **5.36** | **6.53** | **5.90** | **4.10** | **4.66** | **4.38** | **5.39** |
| Terpinen-4-ol | 1622 | 1184 | | 0.03 | 0.03 | 0.06 | 0.04 | | 0.03 | 0.33 | 0.11 | 0.15 | 0.02 | 0.03 | 0.11 | 0.02 | 0.16 | 0.08 |
| *cis*-α-Necrodyl Acetate | 1629 | 1302 | 1.38 | 1.39 | 1.57 | **1.92** | 1.73 | 1.58 | 1.17 | 1.60 | 1.35 | 1.56 | 1.76 | 1.66 | 1.16 | 1.22 | 1.57 | 1.51 |
| Arbozol | 1636 | 1296 | 1.31 | **2.57** | 1.62 | 1.67 | **2.43** | 1.10 | 1.89 | **2.24** | **2.17** | **2.14** | 1.33 | **1.81** | 1.45 | 1.60 | **2.00** | 1.78 |
| Valerena-4,7(11)-diene | 1637 | 1455 | | | | | | | | | | | | | | 0.08 | | |
| Sabina Ketone | 1645 | 1157 | | | | | | | | 0.03 | | | | 0.01 | 0.03 | 0.08 | 0.02 | |
| Benzeneacetaldehyde | 1649 | 1049 | 0.02 | | | | | 0.04 | | | | | | 0.05 | | | | |
| Myrtenal | 1651 | 1197 | 0.03 | | | | | 0.04 | | | | 0.13 | | 0.04 | | | | |
| Unknown VIII | 1654 | 1326 | | 0.62 | 0.71 | 0.63 | 0.72 | | 0.70 | 0.64 | 0.60 | 0.69 | 0.79 | | 0.68 | 0.69 | 0.66 | 0.56 |
| *cis*-Verbenol | 1660 | 1141 | | | | | | | | | | | | | | | | |
| *trans*-Muurola-3,5-diene | 1661 | 1453 | | | | | | | | | | | | | | | | |
| Alloaromadendrene | 1664 | 1471 | 0.24 | 0.16 | 0.18 | | 0.11 | 0.16 | 0.21 | 0.19 | 0.23 | 0.26 | 0.26 | 0.29 | 0.20 | 0.22 | | 0.24 |

**Table S2c.** (Continued).

| | | | 9 | | 10 | | | | | 11 | | | | | | | | |
|---|---|---|---|---|---|---|---|---|---|---|---|---|---|---|---|---|---|---|
| **Components** | **RI-WAX** | **RI-HP5** | **40** | **41** | **42** | **43** | **44** | **45** | **46** | **47** | **48** | **49** | **50** | **51** | **52** | **53** | **54** | **55** |
| 5-Methylene-2,3,4,4-tetrame-2-Cyclopentenone | 1668 | 1187 | **2.74** | **3.17** | **3.38** | **2.56** | **2.99** | **2.69** | **3.41** | **2.37** | **3.51** | **2.64** | **2.43** | **3.60** | **3.16** | **3.99** | **3.11** | **3.38** |
| Lavandulol | 1669 | 1165 | | | 0.40 | 0.25 | 0.24 | 0.35 | 0.46 | 0.21 | 0.19 | 0.21 | 0.23 | 0.32 | 0.22 | 0.26 | 0.18 | 0.29 |
| Unknown XIX | 1673 | | | 0.59 | 0.69 | 0.81 | 0.70 | | 0.69 | 0.63 | 0.73 | 0.92 | 1.00 | | 0.68 | 0.70 | | 0.72 |
| *trans*-α-Necrodol | 1679 | 1172 | **8.21** | **6.17** | **5.30** | **6.71** | **7.49** | **11.12** | **11.80** | **6.56** | **6.76** | **7.42** | **8.97** | **10.22** | **8.97** | **10.89** | **5.92** | **9.88** |
| *trans*-Cadina-1(6),4-diene | 1682 | 1477 | 0.13 | 0.11 | 0.07 | | 0.09 | 0.08 | 0.10 | 0.08 | 0.09 | | | 0.09 | 0.07 | 0.12 | 0.08 | 0.08 |
| *trans*-Verbenol | 1692 | 1151 | | 0.87 | 0.21 | 0.32 | 0.30 | | 0.66 | 0.23 | 0.31 | 0.22 | 0.24 | | 0.11 | 0.12 | 0.85 | 0.47 |
| α-Humulene | 1694 | 1454 | | 0.04 | 0.05 | | 0.03 | | 0.08 | 0.05 | | | | 0.09 | 0.07 | 0.11 | | |
| β-Acoradiene | 1694 | 1466 | | | | | | | | | | | | | | | | |
| α-Terpineol | 1696 | 1195 | 0.05 | 0.05 | 0.04 | 0.13 | 0.04 | 0.09 | 0.02 | 0.29 | 0.12 | 0.25 | 0.06 | 0.14 | 0.19 | 0.13 | 0.17 | 0.12 |
| γ-Muurolene | 1700 | 1485 | | 0.04 | 0.04 | 0.04 | 0.02 | | 0.03 | 0.03 | | 0.03 | | | 0.03 | 0.05 | 0.04 | 0.04 |
| Heptadecane | 1700 | 1700 | | | | | | 0.05 | | | | | | | | | 0.04 | |
| Myrtenyl Acetate | 1702 | 1329 | | 0.13 | 0.06 | 0.10 | 0.09 | 0.11 | 0.26 | 0.85 | 0.15 | 0.38 | 0.42 | 0.24 | 0.09 | 0.07 | 0.22 | 0.14 |
| Ledene | 1708 | 1485 | | 0.07 | 0.05 | | 0.05 | | 0.08 | | 0.04 | 0.04 | | | | 0.09 | 0.05 | 0.04 |
| α-Amorphene | 1711 | 1506 | | | | | | | | | | | | | | | 0.26 | |
| Borneol | 1713 | 1167 | | | | | | | | | | | | | | | | |
| Neryl Acetate | 1723 | 1361 | 0.71 | 0.42 | 0.59 | 0.55 | 0.63 | 0.62 | 0.58 | 0.24 | 0.51 | 0.41 | 0.77 | 0.49 | | | 0.51 | 0.51 |
| 10-Epizonarene | 1725 | 1506 | | | | | | | | | | | | | | | | |
| *cis*-Muurola-4(14),5-diene | 1735 | 1466 | | | | | | | | | | | | | | | 0.23 | |
| *cis*-α-Necrodol | 1735 | 1150 | 1.66 | 1.58 | **1.97** | 1.70 | 1.63 | **2.00** | **2.00** | 1.52 | 1.68 | 1.34 | 1.88 | **1.81** | **1.85** | **1.96** | 1.83 | **1.98** |
| Eremophilene | 1735 | 1486 | | | | 0.22 | | 0.39 | | | | 0.10 | | | | | 0.31 | |
| Valencene | 1736 | 1409 | | | | | | | | | | | | | | | | |
| Verbenone | 1736 | 1208 | | 0.60 | 0.32 | 0.36 | 0.26 | 0.27 | 0.33 | 0.26 | 0.12 | 0.33 | 0.26 | 0.17 | | | 0.31 | 0.18 |
| α-Selinene | 1737 | 1501 | | | 0.15 | 0.09 | 0.08 | | 0.10 | 0.05 | 0.09 | 0.07 | | | 0.07 | 0.13 | 0.09 | 0.09 |
| Unknown X | 1739 | 1173 | 1.52 | | | | | **1.87** | | | | | | **1.87** | | | | |
| α-Muurolene | 1746 | 1501 | 0.30 | | | | | 0.16 | | | | | | 0.27 | | | | |

**Table S2c.** (Continued).

| | | | 9 | | 10 | | | | | 11 | | | | | | | | |
|---|---|---|---|---|---|---|---|---|---|---|---|---|---|---|---|---|---|---|
| **Components** | **RI-WAX** | **RI-HP5** | **40** | **41** | **42** | **43** | **44** | **45** | **46** | **47** | **48** | **49** | **50** | **51** | **52** | **53** | **54** | **55** |
| β-Selinene | 1746 | 1502 | 0.16 | 0.22 | 0.35 | 0.29 | 0.17 | 0.31 | 0.20 | 0.28 | 0.22 | 0.14 | | 0.20 | 0.33 | 0.20 | 0.35 | 0.20 |
| Geranyl Acetate | 1758 | 1380 | 0.08 | | 0.15 | 0.21 | 0.22 | 0.19 | 0.27 | 0.18 | 0.18 | 0.21 | 0.36 | 0.16 | 0.09 | 0.08 | 0.11 | 0.18 |
| (E,E) α Famesene | 1760 | 1508 | | | | | | | | | | | | | | | | |
| Carvone | 1762 | 1246 | | 0.06 | 0.04 | 0.06 | 0.04 | | 0.03 | 0.04 | 0.02 | | | 0.02 | 0.03 | 0.02 | 0.04 | |
| *trans*-Linalool Oxide (Pyranoid) | 1767 | 1170 | 0.03 | 0.09 | 0.06 | 0.07 | 0.04 | 0.04 | 0.06 | 0.03 | | | 0.04 | | 0.03 | 0.02 | 0.04 | 0.03 |
| δ-Cadinene | 1776 | 1524 | 0.63 | 0.99 | 0.72 | 0.36 | 0.32 | 0.21 | 0.67 | 0.62 | 0.83 | 0.66 | 0.49 | 0.93 | 0.62 | 1.15 | 0.98 | 0.87 |
| Unknown XI | 1793 | 1173 | | **2.00** | 1.85 | 1.11 | 1.78 | | 1.61 | 1.55 | 1.69 | 1.28 | 1.16 | | 1.81 | 1.76 | 1.63 | **2.10** |
| γ-Cadinene | 1782 | 1515 | | | | 0.31 | | | | 0.32 | 0.42 | 0.29 | 0.51 | | 0.46 | 0.68 | 0.47 | 0.45 |
| 1,7,7-Trimethylbicyclo[2.2.1]hept-5-en-2-ol | 1785 | 1120 | | | | | | | | 0.11 | | | | | 0.07 | 0.06 | | |
| Myrtenol | 1798 | 1202 | | | | | | | | | | | | | | | | |
| *p*-Methyl Acetophenone | 1800 | 1183 | | 0.05 | 0.05 | | 0.03 | | 0.03 | | | | | | | | | |
| Eudesma-3,7(11)-diene | 1805 | 1532 | 0.66 | 1.13 | **2.29** | 0.59 | 0.47 | 1.18 | 1.09 | 0.97 | 0.95 | 0.67 | 0.14 | 0.67 | 0.91 | 0.97 | 1.77 | 0.56 |
| α-Cadinene | 1817 | 1538 | | | | | | | | | | | | | | | | |
| *trans*-Carveol | 1822 | 1223 | | | | 0.07 | | | | | | | | | | | | |
| Phenethyl Acetate | 1828 | 1257 | | | | | | | | | | | | | | | | |
| *p*-Cymen-8-ol | 1839 | 1189 | 0.15 | 0.07 | 0.08 | 0.05 | 0.18 | 0.07 | 0.20 | 0.03 | | | | | 0.02 | 0.03 | 0.03 | |
| *cis*-Carveol | 1852 | 1232 | | 0.10 | 0.06 | | 0.08 | | 0.05 | 0.07 | 0.02 | | | | 0.05 | 0.03 | 0.06 | |
| Unknown XII | 1852 | 1393 | | | | | | | | | 1.09 | | | | | | | |
| Germacrene B | 1853 | 1557 | | | | | | 0.04 | | | | | | | | | | |
| *trans*-Calamenene | 1857 | 1527 | | | | 0.22 | | | 0.04 | 0.05 | | | 0.11 | | 0.04 | 0.07 | 0.13 | |
| Unknown XIII | | 1860 | | **2.00** | 1.80 | 1.23 | **2.84** | | **2.33** | **2.06** | **3.04** | **2.48** | **2.56** | | **2.59** | **3.56** | **2.46** | **2.54** |
| Unknown XIV | | 1861 | **3.60** | | | 0.87 | | 0.72 | | | | | | 1.15 | | | | |
| *meta*-Cymen-8-ol | 1861 | 1183 | | 0.18 | 0.05 | | 0.11 | | 0.17 | 0.12 | | | | | 0.10 | 0.22 | 0.25 | |
| Unknown XV | 1862 | 1393 | | 0.87 | 0.84 | | 1.04 | | 0.94 | 1.02 | | | | | 0.78 | 0.84 | 0.83 | |
| Unknown XVI | | 1870 | **2.83** | 1.65 | 1.54 | | **2.20** | 0.55 | 1.85 | 1.66 | **2.43** | **1.94** | **1.98** | 0.82 | **2.06** | **2.87** | 1.93 | 1.97 |

**Table S2c.** (Continued).

| Components | RI-WAX | RI-HP5 | 9 | | 10 | | | | | 11 | | | | | | | | |
|---|---|---|---|---|---|---|---|---|---|---|---|---|---|---|---|---|---|---|
| | | | **40** | **41** | **42** | **43** | **44** | **45** | **46** | **47** | **48** | **49** | **50** | **51** | **52** | **53** | **54** | **55** |
| Cubebol Isomer I | 1903 | 1516 | 0.77 | | | | | 0.32 | | | 0.72 | | | 0.64 | | | 0.87 | |
| *epi*-Cubebol | 1903 | 1498 | 1.08 | 0.62 | 0.39 | 0.32 | 0.35 | 0.67 | 0.40 | 0.37 | 0.52 | 0.37 | 0.70 | 0.93 | 0.52 | 0.82 | 0.56 | 0.50 |
| 10,11-Epoxycalamenene | 1903 | 1498 | | | | | | | | | | | | | | | | |
| *cis*-Muurol-5-en-4-β-ol | 1905 | 1548 | | | | | | | | | | | | | | | | |
| Unknown XVII | 1946 | 1564 | 1.01 | | | | | 0.55 | | | | | | 0.76 | 0.54 | | | |
| α-Calacorene | 1944 | 1542 | | 0.09 | 0.07 | 0.08 | 0.07 | | 0.06 | 0.07 | 0.12 | 0.12 | 0.14 | | 0.06 | 0.11 | 0.12 | 0.11 |
| Palustrol | 1958 | 1568 | 0.10 | 0.15 | 0.10 | 0.05 | | 0.06 | 0.16 | 0.05 | 0.06 | | | 0.07 | 0.05 | 0.08 | 0.06 | |
| *trans*-Muurol-5-en-4-β-ol | 1958 | | | 0.31 | 0.30 | 0.31 | | | 0.27 | 0.29 | 0.47 | 0.27 | 0.56 | | 0.56 | 0.85 | 0.51 | 0.47 |
| n-Dodecanol | 1975 | 1476 | | | | | 0.20 | | | | | | | | | | | |
| Unknown XVIII | 1996 | 1564 | | 0.63 | 0.52 | 0.50 | 0.66 | | 0.59 | 0.51 | 0.57 | 0.57 | 0.73 | | | 0.83 | 0.59 | 0.60 |
| Caryophyllene Oxide | 2014 | 1578 | 0.60 | 0.46 | 0.35 | 0.43 | 0.40 | 0.27 | 0.36 | 0.33 | 0.38 | 0.32 | 0.70 | 0.39 | 0.40 | 0.59 | 0.51 | 0.38 |
| Unknown XIX | 2059 | 1578 | 1.53 | | | | | 0.59 | | | | | | 1.17 | | 0.35 | | |
| Ledol | 2066 | 1574 | 0.81 | 0.57 | 0.33 | 0.46 | 0.44 | 0.40 | 0.22 | 0.31 | 0.51 | 0.43 | 0.59 | 0.57 | 0.34 | 0.19 | 0.42 | 0.48 |
| 1,10-Di-*epi*-Cubenol | 2099 | 1614 | | 0.17 | 0.08 | 0.31 | 0.20 | | 0.10 | 0.10 | | | 0.26 | | 0.07 | | 0.14 | 0.13 |
| Viridiflorol | 2103 | 1610 | **3.24** | **2.96** | 1.68 | 1.68 | **2.16** | **1.89** | **2.03** | 1.62 | 2.00 | 1.58 | **2.50** | **2.38** | 1.78 | **2.81** | **2.13** | **1.98** |
| 1-*epi*-Cubenol | 2107 | 1613 | 0.82 | 0.42 | 0.28 | 0.28 | 0.41 | 0.42 | 0.33 | 0.30 | 0.38 | 0.31 | 0.43 | 0.46 | 0.31 | 0.53 | 0.40 | 0.36 |
| Unknown XX | 2115 | 1577 | | 0.91 | 0.72 | 0.50 | 0.83 | | 0.80 | 0.69 | 0.76 | 0.83 | 1.04 | | 0.73 | 1.33 | 0.79 | 1.01 |
| Spathulenol | 2149 | 1572 | | | | | | | | | | | | 0.05 | | | | |
| Copaborneol | 2199 | 1613 | 1.58 | 1.38 | 0.79 | 0.82 | 1.07 | 0.84 | 0.99 | 0.77 | 1.00 | 0.78 | | 1.05 | 0.87 | 1.48 | 1.09 | 0.96 |
| (E)-1-(6,10-Dimethylundec-5-en-2-yl)-4-Methylbenzene | 2212 | | | | | | | | | 0.11 | | | | 0.18 | 0.11 | 0.12 | 0.07 | |
| Pentylcurcumene | 2212 | | | | | | | | | | | | | | | | | 0.20 |
| T-Muurolol | 2215 | 1645 | 0.58 | 0.23 | 0.08 | 0.20 | 0.25 | 0.24 | 0.23 | 0.17 | 0.34 | 0.32 | 0.36 | 0.37 | 0.20 | 0.24 | 0.18 | 0.24 |
| α-Muurolol | 2224 | 1649 | 0.14 | 0.15 | 0.16 | 0.08 | 0.18 | 0.10 | 0.06 | 0.17 | | 0.04 | | 0.10 | 0.16 | 0.27 | | |
| Cadalene | 2241 | 1670 | 0.11 | | | 0.11 | | 0.08 | | | 0.08 | 0.07 | 0.10 | 0.07 | | 0.10 | 0.09 | 0.08 |
| α-Cadinol | 2249 | 1652 | | | 0.91 | **3.27** | 0.24 | 0.28 | 0.02 | | | | | | | | 0.12 | |

**Table S2c.** (Continued).

| | | | 9 | | 10 | | | | | 11 | | | | | | | | |
|---|---|---|---|---|---|---|---|---|---|---|---|---|---|---|---|---|---|---|
| **Components** | **RI-WAX** | **RI-HP5** | **40** | **41** | **42** | **43** | **44** | **45** | **46** | **47** | **48** | **49** | **50** | **51** | **52** | **53** | **54** | **55** |
| β-Eudesmol | 2251 | 1656 | | | | | | 0.06 | | | | | | | | | | |
| *neo*-Intermedeol | 2264 | 1661 | | | | | | | | | | | | | | | | |
| (E)-1-(6,10-Dimethylundec-5,9-dien-2-yl)-4-Methylbenzene | 2293 | | | | | | | | | | | | | | | | 0.09 | |
| Pentenylcurcumene | 2293 | 1970 | | | | | | | | | | | | | | | | |
| Eudesm-7(11)-en-4-ol | 2312 | 1696 | 0.15 | | | 0.15 | | 0.28 | | | 0.19 | | | 0.15 | | | 0.25 | 0.11 |
| *cis*-14-nor-Muurol-5-en-4-one | 2329 | 1696 | | | | 0.34 | | | | | | | | | | | | |
| Vetiveryl Acetate Isomer | 2342 | 1872 | | | | | | | | | | | | | | | | |
| α-Cyperone | 2370 | 1706 | | | | | | 0.06 | | | | | | | | | | |
| Phenethyl Phenylacetate | 2817 | 1915 | | | | | | | | | | | | | | | | |
| **% Identification** | | | 83.17 | 83.05 | 85.86 | 89.20 | 84.30 | 81.15 | 90.58 | 91.18 | 88.98 | 92.24 | 90.25 | 82.62 | 91.57 | 88.82 | 85.97 | 90.00 |
| **Yield (g/kg)** | | | 1.97 | 2.64 | 2.20 | 3.23 | 2.66 | 3.27 | 2.57 | 4.22 | 2.46 | 4.25 | 3.16 | 1.79 | 2.86 | 2.08 | 2.53 | 2.48 |
| Monoterpene hydrocarbons | | | 5.91 | 6.46 | 5.94 | 3.15 | 6.89 | 6.93 | 6.20 | 6.17 | 4.78 | 5.79 | 3.49 | 4.79 | 4.64 | 4.66 | 4.76 | 5.50 |
| Oxygen-containing monoterpenes | | | 47.26 | 44.70 | 50.88 | 60.01 | 48.66 | 54.53 | 56.12 | 59.65 | 52.88 | 61.51 | 60.67 | 53.37 | 59.33 | 47.52 | 49.03 | 55.89 |
| Sesquiterpene hydrocarbons | | | 3.61 | 3.71 | 4.86 | 2.74 | 1.95 | 3.28 | 3.49 | 3.28 | 3.83 | 3.06 | 2.48 | 3.88 | 3.60 | 5.57 | 5.97 | 3.61 |
| Oxygen-containing sesquiterpenes | | | 9.87 | 7.42 | 5.45 | 8.36 | 5.70 | 5.89 | 5.17 | 4.48 | 6.57 | 4.42 | 6.10 | 7.16 | 5.26 | 7.86 | 7.24 | 5.61 |
| Unknown | | | 4.63 | 6.83 | 5.78 | 5.28 | 6.40 | 4.58 | 6.20 | 5.55 | 6.43 | 5.47 | 4.70 | 6.06 | 5.41 | 6.63 | 6.04 | 6.11 |
| Other | | | 11.89 | 13.93 | 12.95 | 9.66 | 14.7 | 5.94 | 13.40 | 12.05 | 14.49 | 11.99 | 12.81 | 7.36 | 13.33 | 16.58 | 12.93 | 13.28 |

**Table S2d.** Chemical composition of *L. stoechas* subsp. *luisieri* (Zone: 12. Sierra Morena de Sevilla, 13. Vegas Bajas, 14. Villuercas).

| | | | **12** | | | | | | | | **13** | **14** | |
|---|---|---|---|---|---|---|---|---|---|---|---|---|---|
| **Components** | **RI-WAX** | **RI-HP5** | **56** | **57** | **58** | **59** | **60** | **61** | **62** | **63** | **64** | **65** | **66** |
| α-Pinene | 1025 | 933 | 1.27 | 1.31 | 1.27 | **1.54** | 1.67 | 0.91 | 1.77 | 1.58 | **2.47** | **3.23** | **2.42** |
| Methyl Butenol | 1043 | | 0.10 | 0.11 | 0.14 | 0.33 | 0.13 | 0.14 | 0.11 | 0.15 | 0.15 | 0.13 | 0.12 |
| α-Fenchene | 1060 | 948 | | | | | | | | | | 0.03 | 0.02 |
| Camphene | 1069 | 953 | 0.05 | 0.15 | 0.11 | 0.05 | 0.13 | 0.06 | 0.08 | 0.10 | 0.10 | 0.21 | 0.11 |
| n-Hexanal | 1090 | 796 | 0.02 | | 0.03 | 0.02 | | 0.03 | 0.02 | | 0.03 | 0.02 | 0.03 |
| β-Pinene | 1114 | 978 | 0.26 | 0.43 | 0.20 | 0.33 | 0.15 | 0.02 | 0.19 | 0.24 | 0.10 | 0.31 | 0.02 |
| Sabinene | 1126 | 972 | 0.11 | 0.17 | | 0.13 | 0.05 | 0.02 | 0.07 | 0.08 | | 0.13 | |
| Verbenene | 1130 | 971 | 0.09 | 0.06 | 0.07 | 0.05 | 0.06 | 0.04 | 0.07 | 0.05 | 0.11 | 0.09 | 0.11 |
| Cymene Isomer | 1133 | 940 | **2.36** | **2.08** | **2.76** | **2.56** | **2.22** | **2.43** | **2.43** | 1.80 | **2.68** | **2.14** | **2.64** |
| p-Xylene | 1146 | 865 | | <0,01 | 0.01 | 0.01 | 0.02 | | 0.01 | <0,01 | <0.01 | | |
| δ-3-Carene | 1158 | 1009 | 0.03 | 0.02 | 0.04 | 0.04 | 0.03 | 0.04 | 0.05 | 0.02 | 0.18 | 0.37 | 0.96 |
| β-Myrcene | 1165 | 991 | 0.06 | 0.10 | 0.06 | 0.07 | 0.06 | 0.03 | 0.05 | 0.07 | 0.06 | 0.14 | 0.09 |
| 3-Methyl-2-Butenal | 1194 | 779 | 0.04 | 0.04 | 0.05 | 0.04 | 0.02 | 0.04 | 0.04 | 0.04 | 0.06 | 0.04 | 0.05 |
| Limonene | 1206 | 1021 | 0.16 | 0.29 | 0.19 | 0.18 | 0.19 | 0.12 | 0.24 | 0.19 | 0.25 | 0.42 | 0.31 |
| 1,8-Cineole | 1222 | 1039 | **23.82** | **24.24** | **16.02** | **16.57** | **10.29** | 1.59 | **13.83** | **21.35** | 0.95 | **19.84** | **2.33** |
| *cis*-β-Ocimene | 1238 | 1035 | 0.40 | 0.66 | 1.08 | 0.82 | 0.79 | 1.10 | 0.49 | 0.56 | 0.87 | 0.47 | 0.73 |
| γ-Terpinene | 1254 | 1058 | | 0.14 | | 0.05 | 0.10 | | 0.10 | 0.12 | | | |
| *trans*-β Ocimene | 1256 | 1046 | | 0.05 | 0.06 | 0.03 | 0.05 | | 0.04 | 0.04 | 0.07 | 0.04 | |
| Prenyl Acetate | 1259 | 920 | 0.05 | 0.04 | 0.06 | 0.02 | 0.04 | 0.04 | 0.05 | 0.04 | 0.07 | 0.04 | 0.05 |
| *p*-Cymene | 1278 | 1025 | 0.21 | 0.17 | 0.16 | 0.11 | 0.13 | 0.06 | 0.14 | 0.17 | 0.11 | 0.21 | 0.19 |
| *m*-Cymene | 1279 | 976 | | | | 0.01 | | | | | 0.01 | 0.03 | 0.09 |
| α-Terpinolene | 1291 | 1086 | 0.08 | 0.06 | 0.07 | 0.06 | 0.04 | | 0.05 | 0.07 | 0.08 | 0.12 | 0.14 |
| Unknown I | | 1281 | 1.42 | 1.26 | 1.53 | | 1.51 | 1.71 | 1.64 | 1.16 | 1.72 | 1.11 | 1.50 |
| 1,2,3,3-Tetramethyl-Cyclopenten-4-one | 1310 | | | 0.26 | | | 0.32 | 0.43 | 0.40 | 0.40 | 0.48 | 0.26 | 0.44 |
| Prenol | 1322 | 772 | | | 0.02 | 0.02 | | 0.02 | | | | | |

**Table S2d.** (Continued).

| | | | 12 | | | | | | | | 13 | 14 | |
|---|---|---|---|---|---|---|---|---|---|---|---|---|---|
| **Components** | **RI-WAX** | **RI-HP5** | **56** | **57** | **58** | **59** | **60** | **61** | **62** | **63** | **64** | **65** | **66** |
| *cis*-3-Hexenyl Acetate | 1328 | 1008 | | 0.01 | | 0.01 | 0.01 | | 0.01 | 0.01 | 0.02 | | 0.02 |
| 6-Methyl-5-Hepten-2-one | 1351 | 986 | | 0.01 | | 0.01 | 0.02 | 0.01 | 0.01 | 0.02 | 0.02 | | 0.02 |
| *trans*-3-Hexenol | 1361 | 849 | | 0.11 | | | 0.10 | | 0.06 | 0.13 | 0.12 | 0.08 | |
| n-Hexanol | 1365 | 861 | 0.03 | 0.02 | 0.03 | | 0.02 | | 0.01 | 0.02 | 0.04 | | |
| Alloocimene | 1384 | 1129 | | | 0.05 | | | 0.05 | | | | | |
| 1-Octen-3-yl Acetate | 1387 | 1086 | | | | | | | | | | | |
| *cis*-3-Hexenol | 1390 | 853 | 0.15 | | 0.19 | 0.09 | | 0.10 | | 0.15 | | 0.13 | 0.18 |
| n-Nonanal | 1406 | 1107 | | 0.04 | 0.08 | | | | | 0.06 | 0.05 | | |
| *trans*-2-Hexenol | 1406 | 864 | 0.04 | 0.03 | | 0.03 | 0.04 | 0.03 | 0.03 | 0.04 | 0.05 | 0.03 | |
| Rosefuran | 1412 | 1104 | | 0.01 | 0.02 | 0.01 | 0.02 | 0.03 | 0.02 | 0.02 | 0.02 | | |
| Fenchone | 1418 | 1090 | 0.34 | **2.07** | | 1.22 | 0.69 | 0.13 | 0.39 | 0.75 | 0.46 | **5.41** | **4.95** |
| Unknown II | 1427 | 1083 | | | | 0.86 | | | | | | | |
| 1-Octen-3-ol | 1441 | 975 | 0.13 | | | | | | | | | 0.13 | 0.13 |
| Acetic Acid | 1453 | 505 | 0.04 | | | | | 0.03 | | | | | |
| *cis*-Linalool Oxide (Furanoid) | 1460 | 1070 | 0.74 | 0.33 | 0.61 | 0.48 | 0.88 | 0.62 | 0.38 | 0.43 | 0.89 | 0.50 | 0.45 |
| Unknown III | 1471 | 1085 | 0.97 | 0.74 | 1.12 | | 0.82 | 0.95 | 1.00 | 0.98 | 1.27 | 0.72 | 1.12 |
| $\alpha$-Cubebene | 1473 | 1351 | | | | | | | | | | | |
| *trans*-Linalool Oxide (Furanoid) | 1484 | 1086 | 0.59 | 0.29 | 0.43 | 0.28 | 0.69 | 0.46 | 0.27 | 0.33 | 0.67 | 0.36 | 0.38 |
| Cyclosativene | 1493 | 1363 | 0.17 | 0.08 | 0.26 | 0.12 | 0.22 | 0.24 | 0.20 | 0.13 | 0.08 | 0.14 | 0.18 |
| $\alpha$-Campholenal | 1509 | 1125 | | 0.03 | | | 0.05 | | 0.07 | 0.07 | 0.12 | 0.10 | 0.11 |
| $\alpha$-Copaene | 1511 | 1379 | 0.13 | 0.08 | 0.11 | 0.10 | 0.40 | 0.35 | 0.23 | 0.08 | 0.07 | 0.34 | 0.20 |
| Camphor | 1541 | 1149 | 0.66 | **3.31** | **1.89** | 0.66 | **2.36** | 0.94 | 0.79 | **2.96** | **2.02** | **3.45** | **2.22** |
| Cyperene | 1541 | 1390 | | 0.08 | | 0.03 | 0.05 | | 0.04 | 0.03 | 0.04 | | |
| $\alpha$-Gurjunene | 1548 | 1407 | | 0.03 | | | 0.06 | | | | 0.05 | | |
| *cis*-Sabinene Hydrate | 1550 | 1105 | | 0.06 | | | 0.05 | | 0.05 | 0.05 | | | |

**Table S2d.** (Continued).

| | | | 12 | | | | | | | | 13 | 14 | |
|---|---|---|---|---|---|---|---|---|---|---|---|---|---|
| **Components** | **RI-WAX** | **RI-HP5** | **56** | **57** | **58** | **59** | **60** | **61** | **62** | **63** | **64** | **65** | **66** |
| Unknown IV | | 1551 | 0.25 | 0.32 | 0.53 | | 0.05 | | 0.13 | 0.35 | 0.42 | 0.35 | 0.68 |
| Unknown V | 1552 | | 0.33 | 0.31 | 0.38 | | 0.35 | 0.30 | 0.42 | 0.53 | 0.53 | 0.24 | 0.42 |
| Linalool | 1553 | 1100 | **2.79** | **1.64** | **2.74** | **2.31** | **4.27** | **2.92** | **1.96** | **2.59** | **4.25** | **2.72** | 1.52 |
| Linalyl Acetate | 1558 | 1255 | 0.10 | 0.14 | | 0.05 | 0.16 | 0.04 | 0.04 | 0.02 | 0.06 | 0.12 | 0.07 |
| Unknown VI | 1575 | 1156 | 0.51 | 0.38 | 0.62 | | 0.46 | 0.60 | 0.47 | 0.44 | 0.36 | 0.46 | 0.72 |
| Fenchol<*exo*> | 1582 | 1112 | | | | | | | | | | 0.05 | |
| Pinocarvone | 1589 | 1164 | 0.14 | | 0.10 | 0.10 | | 0.04 | | | | 0.15 | |
| γ-Amorphene | 1592 | 1490 | | | | 0.02 | | | | | | | |
| Bornyl Acetate | 1599 | 1288 | | 0.11 | | 0.04 | 0.13 | 0.07 | <0,01 | 0.05 | 0.09 | 0.18 | 0.15 |
| *trans*-α-Necrodyl Acetate | 1606 | 1280 | **15.19** | **19.48** | **18.24** | **20.33** | **20.68** | **23.76** | **19.60** | **12.16** | **20.90** | **14.41** | **18.49** |
| Hotrienol | 1609 | 1108 | | 0.07 | | | 0.05 | | 0.09 | 0.11 | | | |
| Unknown VII | 1609 | 1326 | | | | 0.67 | | | | | | | |
| *trans*-β Caryophyllene | 1617 | 1450 | 0.15 | 0.14 | 0.18 | 0.23 | 0.29 | 0.26 | 0.34 | 0.24 | 0.27 | 0.24 | 0.30 |
| Lavandulyl Acetate | 1619 | 1284 | **4.15** | **3.50** | **4.93** | **3.75** | **3.88** | **3.98** | **4.24** | **3.11** | **4.28** | **3.00** | **4.69** |
| Terpinen-4-ol | 1622 | 1184 | 0.17 | 0.20 | 0.13 | 0.17 | 0.09 | 0.01 | 0.12 | 0.40 | 0.03 | 0.27 | 0.08 |
| *cis*-α-Necrodyl Acetate | 1629 | 1302 | 1.38 | 0.97 | 1.67 | **1.54** | 1.58 | 1.70 | 1.49 | 0.84 | 1.72 | 1.02 | 1.22 |
| Arbozol | 1636 | 1296 | 1.24 | **1.62** | **2.20** | 1.39 | 2.01 | 1.90 | 1.95 | 1.13 | **2.43** | 1.56 | 1.42 |
| Valerena-4,7(11)-diene | 1637 | 1455 | | | | | | | | | | | |
| Sabina Ketone | 1645 | 1157 | 0.05 | 0.05 | 0.03 | 0.03 | 0.02 | | 0.03 | 0.04 | | | |
| Benzeneacetaldehyde | 1649 | 1049 | | | | 0.03 | | | | | | | |
| Myrtenal | 1651 | 1197 | | | | 0.08 | | | | | | | |
| Unknown VIII | 1654 | 1326 | 0.44 | 0.53 | 0.52 | | 0.64 | 0.61 | 0.62 | 0.29 | 0.71 | 0.35 | 0.45 |
| *cis*-Verbenol | 1660 | 1141 | | | | | | | | | | | |
| *trans*-Muurola-3,5-diene | 1661 | 1453 | | | | | | | | | | | |
| Alloaromadendrene | 1664 | 1471 | 0.12 | 0.07 | 0.16 | 0.13 | 0.21 | 0.24 | 0.19 | 0.10 | 0.11 | | |

**Table S2d.** (Continued).

| Components | RI-WAX | RI-HP5 | 12 | | | | | | | | 13 | 14 | |
|---|---|---|---|---|---|---|---|---|---|---|---|---|---|
| | | | 56 | 57 | 58 | 59 | 60 | 61 | 62 | 63 | 64 | 65 | 66 |
| 5-Methylene-2,3,4,4-tetrame-2-Cyclopentenone | 1668 | 1187 | **3.15** | **2.36** | **3.76** | **2.31** | **3.26** | **3.61** | **3.13** | **2.50** | **3.52** | 1.89 | **3.15** |
| Lavandulol | 1669 | 1165 | 0.23 | 0.20 | 0.20 | | 0.21 | 0.29 | 0.28 | 0.52 | 0.04 | 0.18 | 0.41 |
| Unknown IX | 1673 | | 0.50 | 0.50 | 0.51 | | 0.63 | 0.75 | 0.61 | 0.35 | 0.67 | 0.48 | 0.65 |
| *trans*-α-Necrodol | 1679 | 1172 | **6.57** | **5.14** | **6.96** | **10.93** | **8.46** | **9.31** | **7.97** | **6.94** | **8.07** | **4.32** | **8.22** |
| *trans*-Cadina-1(6),4-diene | 1682 | 1477 | 0.07 | 0.06 | | 0.03 | 0.08 | 0.09 | 0.08 | 0.06 | 0.06 | 0.07 | 0.09 |
| *trans*-Verbenol | 1692 | 1151 | 0.67 | 0.18 | 0.49 | | 0.27 | 0.14 | 0.68 | 0.19 | 0.39 | 0.58 | 0.46 |
| α-Humulene | 1694 | 1454 | | 0.02 | | | 0.06 | | 0.06 | 0.03 | 0.04 | | |
| β-Acoradiene | 1694 | 1466 | | | | | | | | | | | |
| α-Terpineol | 1696 | 1195 | 0.22 | 0.44 | 0.20 | 0.34 | 0.13 | 0.02 | 0.17 | 0.26 | 0.48 | 0.44 | 0.11 |
| γ-Muurolene | 1700 | 1485 | 0.02 | 0.02 | | | 0.03 | 0.04 | 0.03 | 0.02 | 0.01 | 0.02 | 0.03 |
| Heptadecane | 1700 | 1700 | | | | | | | | | | | |
| Myrtenyl Acetate | 1702 | 1329 | 0.27 | 0.35 | 0.17 | 0.22 | 0.27 | 0.44 | 0.01 | 0.27 | 0.03 | 0.51 | 0.17 |
| Ledene | 1708 | 1485 | 0.04 | | | | 0.05 | 0.04 | 0.06 | | 0.04 | | 0.04 |
| α-Amorphene | 1711 | 1506 | | | | | | | | | | | |
| Borneol | 1713 | 1167 | | | | | | | | | | | |
| Neryl Acetate | 1723 | 1361 | 0.20 | 0.19 | 0.24 | 0.24 | 0.46 | 0.65 | 0.43 | 0.25 | 0.48 | 0.15 | 0.34 |
| 10-Epizonarene | 1725 | 1506 | | | | | | | | | | | |
| *cis*-Muurola-4(14),5-diene | 1735 | 1466 | | | | | | | | | | 0.07 | 0.05 |
| *cis*-α-Necrodol | 1735 | 1150 | **1.57** | 1.29 | 1.52 | **1.79** | 1.66 | **1.99** | **1.96** | 1.69 | 1.93 | 1.27 | 2.03 |
| Eremophilene | 1735 | 1486 | | | | | | 0.06 | | | | | |
| Valencene | 1736 | 1409 | | | | | | | | | | | |
| Verbenone | 1736 | 1208 | 0.31 | 0.24 | 0.25 | 0.23 | 0.25 | 0.16 | 0.32 | 0.26 | 0.39 | 0.27 | 0.40 |
| α-Selinene | 1737 | 1501 | 0.05 | 0.06 | 0.09 | | | 0.05 | 0.04 | 0.08 | 0.11 | 0.09 | 0.15 |
| Unknown X | 1739 | 1173 | | | | **1.86** | | | | | | | |
| α-Muurolene | 1746 | 1501 | | | | 0.13 | | | | | | | |

**Table S2d.** (Continued).

| Components | RI-WAX | RI-HP5 | 12 | | | | | | | | 13 | 14 | |
|---|---|---|---|---|---|---|---|---|---|---|---|---|---|
| | | | 56 | 57 | 58 | 59 | 60 | 61 | 62 | 63 | 64 | 65 | 66 |
| β-Selinene | 1746 | 1502 | 0.17 | 0.25 | 0.25 | 0.19 | 0.18 | 0.10 | 0.29 | 0.41 | 0.22 | 0.20 | 0.38 |
| Geranyl Acetate | 1758 | 1380 | 0.13 | 0.21 | 0.13 | 0.24 | 0.11 | 0.10 | 0.08 | 0.09 | 0.21 | 0.22 | 0.15 |
| (E,E) α Famesene | 1760 | 1508 | | | | | | | | | | | |
| Carvone | 1762 | 1246 | | 0.05 | | 0.04 | 0.04 | 0.03 | 0.04 | 0.04 | 0.05 | 0.08 | |
| *trans*-Linalool Oxide (Pyranoid) | 1767 | 1170 | | 0.03 | | | 0.06 | 0.02 | 0.03 | 0.03 | 0.06 | | |
| δ-Cadinene | 1776 | 1524 | 0.76 | 0.45 | 0.75 | 0.31 | 0.68 | 1.10 | 0.60 | 0.45 | 0.47 | 0.46 | 0.69 |
| Unknown XI | 1793 | 1173 | 1.40 | 1.14 | 1.61 | | 1.51 | 1.88 | **2.20** | **1.81** | 1.69 | 1.36 | **2.12** |
| γ-Cadinene | 1782 | 1515 | 0.26 | | 0.27 | | | 0.59 | | 0.10 | | | |
| 1,7,7-Trimethylbicyclo[2.2.1]hept-5-en-2-ol | 1785 | 1120 | 0.09 | | 0.07 | | | | | 0.11 | | | |
| Myrtenol | 1798 | 1202 | | | | | | | | | | | |
| *p*-Methyl Acetophenone | 1800 | 1183 | | | | | | | | | 0.04 | | |
| Eudesma-3,7(11)-diene | 1805 | 1532 | 0.40 | 0.83 | 1.39 | 0.74 | 0.12 | 0.20 | 0.53 | 1.02 | 0.98 | 0.97 | 1.43 |
| α-Cadinene | 1817 | 1538 | | | | | | | | | | | |
| *trans*-Carveol | 1822 | 1223 | | | | | | | | | | | |
| Phenethyl Acetate | 1828 | 1257 | | | | | | | | | | | |
| *p*-Cymen-8-ol | 1839 | 1189 | | 0.02 | | 0.01 | 0.05 | | 0.02 | 0.03 | 0.06 | 0.12 | 0.46 |
| *cis*-Carveol | 1852 | 1232 | 0.06 | 0.05 | | | 0.08 | 0.03 | 0.09 | 0.07 | 0.10 | | |
| Unknown XII | 1852 | 1393 | | | | | | | | | | | |
| Germacrene B | 1853 | 1557 | | | | | | | | | | | |
| *trans*-Calamenene | 1857 | 1527 | | 0.03 | | | 0.06 | | 0.06 | 0.10 | 0.11 | 0.07 | |
| Unknown XIII | | 1860 | **1.61** | 1.46 | 1.62 | | **2.44** | **3.71** | **2.25** | 1.23 | 1.65 | 1.31 | 1.61 |
| Unknown XIV | | 1861 | | | | 0.66 | | | | | | | |
| *meta*-Cymen-8-ol | 1861 | 1183 | | 0.13 | | | 0.12 | | 0.12 | 0.11 | 0.04 | | |
| Unknown XV | 1862 | 1393 | | 0.79 | | | 0.79 | | 0.71 | 0.48 | 1.04 | | |
| Unknown XVI | | 1870 | 1.22 | 1.16 | 1.29 | 0.47 | 1.94 | **2.89** | 1.79 | 0.99 | 1.27 | 1.05 | 1.28 |

**Table S2d.** (Continued).

| | | | 12 | | | | | | | | 13 | 14 | |
|---|---|---|---|---|---|---|---|---|---|---|---|---|---|
| **Components** | **RI-WAX** | **RI-HP5** | **56** | **57** | **58** | **59** | **60** | **61** | **62** | **63** | **64** | **65** | **66** |
| Cubebol Isomer I | 1903 | 1516 | | | | 0.27 | | 0.77 | | | | | 0.26 |
| *epi*-Cubebol | 1903 | 1498 | 0.30 | 0.22 | 0.29 | 0.49 | 0.56 | 0.68 | 0.62 | 0.32 | 0.23 | 0.22 | 0.34 |
| 10,11-Epoxycalamenene | 1903 | 1498 | | | | | | | | | | | |
| *cis*-Muurol-5-en-4-β-ol | 1905 | 1548 | | | | | | | | | | | |
| Unknown XVII | 1946 | 1564 | | | | 0.49 | | | | | | | |
| α-Calacorene | 1944 | 1542 | 0.09 | 0.04 | 0.09 | | 0.08 | 0.13 | 0.07 | 0.08 | 0.09 | 0.12 | 0.12 |
| Palustrol | 1958 | 1568 | 0.06 | 0.03 | | 0.04 | 0.06 | 0.08 | 0.06 | 0.05 | 0.13 | | |
| *trans*-Muurol-5-en-4-β-ol | 1958 | | 0.22 | 0.17 | 0.28 | | 0.39 | 0.61 | 0.45 | 0.37 | 0.20 | | 0.18 |
| n-Dodecanol | 1975 | 1476 | | | | | | | | | | | |
| Unknown XVIII | 1996 | 1564 | 0.43 | 0.33 | 0.42 | | 0.59 | 0.77 | 0.65 | 0.41 | 0.43 | 0.37 | 0.56 |
| Caryophyllene Oxide | 2014 | 1578 | 0.33 | 0.20 | 0.31 | 0.27 | 0.39 | 0.48 | 0.44 | 0.35 | 0.35 | 0.29 | 0.35 |
| Unknown XIX | 2059 | 1578 | | | | 0.59 | | | | | | | |
| Ledol | 2066 | 1574 | 0.51 | 0.14 | 0.47 | 0.29 | 0.52 | 0.66 | 0.48 | 0.21 | 0.38 | 0.37 | 0.51 |
| 1,10-Di-*epi*-Cubenol | 2099 | 1614 | | 0.06 | | | 0.10 | 0.26 | 0.23 | 0.43 | 0.30 | 0.13 | 0.15 |
| Viridiflorol | 2103 | 1610 | **2.07** | 1.25 | **1.91** | 1.46 | **2.13** | **2.72** | **2.29** | **1.82** | 2.01 | 1.41 | 1.95 |
| 1-*epi*-Cubenol | 2107 | 1613 | 0.33 | 0.20 | 0.31 | 0.01 | 0.35 | 0.46 | 0.42 | 0.31 | 0.33 | 0.25 | 0.37 |
| Unknown XX | 2115 | 1577 | 0.53 | 0.45 | 0.44 | | 0.97 | 1.20 | 0.88 | 0.45 | 0.39 | 0.35 | 0.60 |
| Spathulenol | 2149 | 1572 | | | | | | | | | | | |
| Copaborneol | 2199 | 1613 | 0.95 | 0.57 | 0.91 | 0.81 | 0.99 | 1.38 | 1.10 | 0.88 | 0.93 | 0.69 | 0.95 |
| (E)-1-(6,10-Dimethylundec-5-en-2-yl)-4-Methylbenzene | 2212 | | | 0.13 | | | | | | | | | |
| Pentylcurcumene | 2212 | | 0.09 | | 0.19 | | | | | | | | |
| T-Muurolol | 2215 | 1645 | 0.22 | 0.12 | 0.28 | 0.21 | 0.23 | 0.43 | 0.24 | 0.22 | 0.23 | 0.16 | 0.16 |
| α-Muurolol | 2224 | 1649 | | 0.15 | | 0.15 | 0.22 | 0.07 | 0.06 | 0.13 | 0.25 | | 0.15 |
| Cadalene | 2241 | 1670 | 0.08 | | 0.08 | 0.06 | 0.06 | 0.10 | | | 0.14 | 0.10 | 0.12 |
| α-Cadinol | 2249 | 1652 | | 0.03 | | | | | | **5.10** | **3.46** | **2.52** | 0.90 |

**Table S2d.** (Continued).

| | | | 12 | | | | | | | | 13 | 14 | |
|---|---|---|---|---|---|---|---|---|---|---|---|---|---|
| **Components** | **RI-WAX** | **RI-HP5** | **56** | **57** | **58** | **59** | **60** | **61** | **62** | **63** | **64** | **65** | **66** |
| β-Eudesmol | 2251 | 1656 | | | | | | | | | | | |
| *neo*-Intermedeol | 2264 | 1661 | | | | | | | | | | | |
| (E)-1-(6,10-Dimethylundec-5,9-dien-2-yl)-4-Methylbenzene | 2293 | | | | | | | | | | | | |
| Pentenylcurcumene | 2293 | 1970 | | | | | | | | | | | |
| Eudesm-7(11)-en-4-ol | 2312 | 1696 | 0.11 | | 0.22 | 0.18 | | 0.06 | | | 0.23 | 0.14 | 0.23 |
| *cis*-14-nor-Muurol-5-en-4-one | 2329 | 1696 | | | | | | | | | | | 0.25 |
| Vetiveryl Acetate Isomer | 2342 | 1872 | | | | | | | | | | | |
| α-Cyperone | 2370 | 1706 | | | | | | | | | | | |
| Phenethyl Phenylacetate | 2817 | 1915 | | | | | | | | | | | |
| **% Identification** | | | 87.82 | 90.24 | 89.12 | 83.87 | 90.99 | 88.35 | 89.75 | 88.51 | 87.03 | 89.19 | 85.09 |
| **Yield (g/kg)** | | | 3.03 | 4.14 | 2.10 | 3.89 | 3.24 | 2.65 | 3.02 | 2.84 | 1.75 | 4.03 | 2.80 |
| Monoterpene hydrocarbons | | | 5.08 | 5.69 | 6.12 | 6.03 | 5.67 | 4.88 | 5.77 | 5.09 | 7.09 | 7.94 | 7.83 |
| Oxygen-containing monoterpenes | | | 60.39 | 64.97 | 57.01 | 61.63 | 58.04 | 49.47 | 55.54 | 56.10 | 48.79 | 59.72 | 49.66 |
| Sesquiterpene hydrocarbons | | | 2.51 | 2.24 | 3.63 | 2.09 | 2.63 | 3.59 | 2.82 | 2.93 | 2.89 | 2.89 | 3.78 |
| Oxygen-containing sesquiterpenes | | | 5.10 | 3.14 | 4.98 | 4.18 | 5.94 | 8.66 | 6.39 | 10.19 | 9.03 | 6.18 | 6.50 |
| Unknown | | | 5.13 | 4.83 | 6.79 | 4.34 | 6.01 | 6.38 | 5.86 | 4.73 | 7.08 | 4.31 | 5.61 |
| Other | | | 9.61 | 9.37 | 10.59 | 5.60 | 12.7 | 15.37 | 13.37 | 9.47 | 12.15 | 8.15 | 11.71 |
