# Supplementary material for: Lavandula stoechas subsp. luisieri (Rozeira) Rozeira: Variability of Chemical Composition of Essential Oil in Wild Populations
Source: Plants (Basel). 2025 Nov 10;14(22):3435. doi: 10.3390/plants14223435 (PMC12656282; doi:10.3390/plants14223435)
Supplement: Supplementary file 1 [file plants-14-03435-s001.zip › Suplement S3. Main components.pdf]

plants

Table S3a. Main components of *L. stoechas* subsp. *luisieri* essential oil (Zone: 1. Alconera. 2. Atlantic coast).

	1				2			
	**μ ± σ**	**min.**	**max.**	**n**	**μ ± σ**	**min.**	**max.**	**n**
α-Pinene	1.65			1	1.15 ± 0.25	0.67	1.45	7
Cymene Isomer	**1.95**			1	1.58 ± 0.97	0.17	2.22	7
1.8-Cineole	**24.13**			1	**9.42 ± 7.56**	1.05	21.80	7
Unknown I					1.62 ± 0.29	1.30	2.01	7
Fenchone	0.45			1	**3.68 ± 4.53**	0.23	12.92	7
Camphor	0.58			1	1.80 ± 1.45	0.23	4.79	7
Linalool	**3.01**			1	**2.24 ± 0.90**	1.09	3.33	7
trans-α-Necrodyl Acetate	**11.11**			1	**17.59 ± 2.56**	15.30	21.69	7
Lavandulyl Acetate	**4.43**			1	**5.07 ± 0.96**	4.15	6.34	7
cis-α-Necrodyl Acetate	1.27			1	1.31 ± 0.37	0.82	1.81	7
Arbozol	1.11			1	1.53 ± 0.16	1.25	1.71	7
5-Methylene-2,3,4,4-tetrame-2-Cyclopentenone	**2.55**			1	**2.64 ± 0.76**	1.83	4.00	7
Trans-α-Necrodol	**7.84**			1	**8.14 ± 2.99**	4.59	12.38	7
cis-α-Necrodol	1.46			1	**1.86 ± 0.33**	1.46	2.29	7
Unknown X	**1.98**			1				
Unknown XI					1.55 ± 0.44	1.13	2.31	7
Eudesma-3,7(11)-diene	0.65			1	1.08 ± 0.32	0.60	1.42	7
Unknown XIII					**2.20 ± 0.91**	1.43	3.60	7
Unknown XIV	**1.93**			1				
Unknown XVI	1.50			1	1.69 ± 0.73	1.10	2.75	7
Viridiflorol	**1.75**			1	**2.22 ± 0.86**	1.12	3.22	7
Unknown XX					1.00 ± 0.53	0.51	1.99	7
Copaborneol	0.83			1	1.14 ± 0.47	0.54	1.69	7
α-Cadinol					0.27 ± 0.32	0.00	0.88	7

Note. μ ± σ: plus or minus one standard deviation from the mean. min.: minimum value; max.: maximum value. n: number of samples.

Table S3b. Main components of *L. stoechas* subsp. *luisieri* essential oil (Zone: 3. Guadiana International. 4. Montes de Toledo).

	**3**				**4**			
	**μ ± σ**	**min.**	**max.**	**n**	**μ ± σ**	**min.**	**max.**	**n**
α-Pinene	**1.84 ± 0.17**	1.72	1.96	2	**3.23 ± 1.08**	1.92	5.36	10
Cymene Isomer	**2.57 ± 0.16**	2.46	2.68	2	**1.79 ± 0.79**	0.17	2.71	10
1.8-Cineole	**18.41 ± 0.28**	18.21	18.60	2	**8.40 ± 4.27**	0.18	14.01	10
Unknown I	0.73 ± 1.03	0.00	1.46	2	1.47 ± 0.17	1.25	1.86	10
Fenchone	0.21 ± 0.24	0.04	0.38	2	**3.61 ± 1.84**	0.78	7.39	10
Camphor	0.95 ± 0.06	0.90	0.99	2	**2.44 ± 1.62**	0.16	6.00	10
Linalool	**2.19 ± 0.32**	1.96	2.41	2	**2.39 ± 0.61**	1.31	3.29	10
trans-α-Necrodyl Acetate	**20.23 ± 0.07**	20.18	20.28	2	**22.8 ± 3.49**	18.17	29.94	10
Lavandulyl Acetate	**4.71 ± 0.81**	4.13	5.28	2	**4.45 ± 0.80**	2.95	5.83	10
cis-α-Necrodyl Acetate	1.42 ± 0.24	1.25	1.59	2	1.35 ± 0.28	0.94	1.76	10
Arbozol	**1.91 ± 0.30**	1.70	2.13	2	1.55 ± 0.42	0.84	2.36	10
5-Methylene-2,3,4,4-tetrame-2-Cyclopentenone	**2.26 ± 0.24**	2.09	2.43	2	1.66 ± 0.32	1.26	2.44	10
Trans-α-Necrodol	**6.59 ± 0.52**	6.22	6.96	2	**9.76 ± 2.06**	7.70	13.57	10
cis-α-Necrodol	1.41 ± 0.07	1.36	1.46	2	1.56 ± 0.29	1.27	2.12	10
Unknown X	0.87 ± 1.22	0.00	1.73	2				
Unknown XI	0.60 ± 0.85	0.00	1.20	2	1.19 ± 0.46	0.57	1.88	10
Eudesma-3,7(11)-diene	0.96 ± 0.47	0.62	1.29	2	1.31 ± 0.73	0.40	2.69	10
Unknown XIII	0.27 ± 0.38	0.00	0.54	2	0.51 ± 0.63	0.00	1.71	10
Unknown XIV	1.02 ± 1.44	0.00	2.04	2				
Unknown XVI	0.94 ± 0.76	0.40	1.48	2	0.38 ± 0.48	0.00	1.31	10
Viridiflorol	1.11 ± 0.78	0.56	1.66	2	0.43 ± 0.44	0.00	1.05	10
Unknown XX	0.09 ± 0.13	0.00	0.18	2	0.20 ± 0.24	0.00	0.66	10
Copaborneol	0.56 ± 0.52	0.19	0.93	2	0.18 ± 0.23	0.00	0.50	10
α-Cadinol	**3.30 ± 4.67**	0.00	6.60	2	**4.82 ± 3.10**	0.25	10.78	10

Note. μ ± σ: plus or minus one standard deviation from the mean. min.: minimum value; max.: maximum value. n: number of samples.

Table S3c. Main components of *L. stoechas* subsp. *luisieri* essential oil (Zone: 5. Rio Tejo. 6. Serra da Gardunha).

	**5**				6			
	**μ ± σ**	**min.**	**max.**	**n**	**μ ± σ**	**min.**	**max.**	**n**
α-Pinene	1.49 ± 0.02	1.47	1.50	2	1.37 ± 0.49	0.57	1.90	5
Cymene Isomer	1.54 ± 1.75	0.30	2.78	2	**1.90 ± 0.80**	0.69	2.51	5
1.8-Cineole	**5.07 ± 6.80**	0.26	9.88	2	**2.54 ± 1.47**	0.41	4.37	5
Unknown I	1.87 ± 0.33	1.63	2.10	2	1.24 ± 0.70	0.00	1.65	5
Fenchone					1.54 ± 1.97	0.00	3.94	5
Camphor	0.82 ± 0.00	0.82	0.82	2	**2.71 ± 2.12**	1.01	6.27	5
Linalool	**3.89 ± 1.05**	3.15	4.63	2	**4.58 ± 0.80**	3.56	5.63	5
trans-α-Necrodyl Acetate	**25.96 ± 2.13**	24.45	27.46	2	**20.44 ± 3.33**	15.90	24.73	5
Lavandulyl Acetate	**5.31 ± 2.34**	3.65	6.96	2	**5.34 ± 0.63**	4.39	6.03	5
cis-α-Necrodyl Acetate	1.54 ± 0.38	1.27	1.81	2	1.35 ± 0.33	0.91	1.62	5
Arbozol	**1.72 ± 0.20**	1.58	1.86	2	1.66 ± 0.47	0.91	2.15	5
5-Methylene-2,3,4,4-tetrame-2-Cyclopentenone	**2.37 ± 0.27**	2.18	2.56	2	**2.71 ± 0.38**	2.20	3.14	5
Trans-α-Necrodol	**10.24 ± 2.94**	8.16	12.32	2	**10.40 ± 0.72**	9.82	11.39	5
cis-α-Necrodol	**1.84 ± 0.46**	1.51	2.16	2	1.80 ± 0.14	1.58	1.95	5
Unknown X					0.40 ± 0.90	0.00	2.02	5
Unknown XI	1.67 ± 0.39	1.39	1.94	2	1.49 ± 0.89	0.00	2.27	5
Eudesma-3,7(11)-diene	0.37 ± 0.17	0.25	0.49	2	1.38 ± 0.42	0.89	1.77	5
Unknown XIII	**2.19 ± 0.07**	2.14	2.24	2	**1.98 ± 1.15**	0.00	2.82	5
Unknown XIV					0.18 ± 0.41	0.00	0.91	5
Unknown XVI	1.69 ± 0.01	1.68	1.69	2	1.64 ± 0.55	0.70	2.02	5
Viridiflorol	**2.05 ± 0.84**	1.45	2.64	2	**2.36 ± 0.42**	1.76	2.89	5
Unknown XX	1.30 ± 0.54	0.92	1.68	2	0.98 ± 0.75	0.00	2.09	5
Copaborneol	1.08 ± 0.43	0.77	1.38	2	1.12 ± 0.20	0.86	1.35	5
α-Cadinol	0.01 ± 0.01	0.00	0.02	2	0.37 ± 0.42	0.00	1.03	5

Note. μ ± σ: plus or minus one standard deviation from the mean. min.: minimum value; max.: maximum value. n: number of samples.

Table S3d. Main components of *L. stoechas* subsp. *luisieri* essential oil (Zone: 7. Serra de Alvelos. 8. Serra de Monchique).

	7				8			
	**μ ± σ**	**min.**	**max.**	**n**	**μ ± σ**	**min.**	**max.**	**n**
α-Pinene	1.59 ± 1.80	0.41	4.77	5	1.00 ± 0.31	0.58	1.50	7
Cymene Isomer	**1.75 ± 0.99**	0.09	2.54	5	**1.92 ± 0.80**	0.42	3.16	7
1.8-Cineole	0.82 ± 0.47	0.20	1.33	5	**6.36 ± 4.96**	0.09	12.86	7
Unknown I	1.32 ± 0.77	0.00	1.91	5	1.27 ± 0.89	0.00	2.04	7
Fenchone	0.22 ± 0.49	0.00	1.10	5	1.91 ± 4.79	0.00	12.76	7
Camphor	**2.67 ± 2.86**	0.92	7.61	5	**2.54 ± 1.99**	0.38	6.42	7
Linalool	**4.91 ± 1.18**	3.72	6.58	5	**3.01 ± 0.79**	2.10	4.55	7
trans-α-Necrodyl Acetate	**18.15 ± 4.07**	13.48	24.32	5	**21.02 ± 4.56**	13.64	26.42	7
Lavandulyl Acetate	**5.15 ± 0.95**	3.73	6.14	5	**4.97 ± 1.00**	3.55	6.35	7
cis-α-Necrodyl Acetate	1.23 ± 0.33	0.84	1.67	5	1.33 ± 0.26	1.04	1.80	7
Arbozol	1.56 ± 0.39	1.02	2.01	5	1.84 ± 0.35	1.57	2.55	7
5-Methylene-2,3,4,4-tetrame-2-Cyclopentenone	**3.15 ± 0.37**	2.85	3.78	5	**3.13 ± 0.76**	2.08	4.13	7
Trans-α-Necrodol	**10.10 ± 2.26**	6.36	11.91	5	**8.24 ± 1.82**	5.16	10.27	7
cis-α-Necrodol	**1.82 ± 0.41**	1.10	2.09	5	1.77 ± 0.29	1.37	2.06	7
Unknown X	0.42 ± 0.93	0.00	2.08	5	0.18 ± 0.47	0.00	1.24	7
Unknown XI	1.28 ± 0.83	0.00	2.11	5	1.20 ± 0.85	0.00	2.02	7
Eudesma-3,7(11)-diene	**1.62 ± 1.10**	0.49	3.24	5	1.18 ± 0.44	0.54	1.76	7
Unknown XIII	**1.88 ± 1.23**	0.00	3.01	5	**2.08 ± 1.11**	0.00	3.21	7
Unknown XIV	0.22 ± 0.48	0.00	1.08	5	0.13 ± 0.35	0.00	0.93	7
Unknown XVI	1.58 ± 0.67	0.81	2.32	5	1.74 ± 0.64	0.72	2.50	7
Viridiflorol	**2.85 ± 0.47**	2.29	3.43	5	**2.33 ± 0.71**	1.24	3.09	7
Unknown XX	0.79 ± 0.56	0.00	1.38	5	0.76 ± 0.38	0.00	1.05	7
Copaborneol	1.45 ± 0.22	1.18	1.78	5	1.14 ± 0.34	0.59	1.43	7
α-Cadinol					0.09 ± 0.13	0.00	0.31	7

Note. μ ± σ: plus or minus one standard deviation from the mean. min.: minimum value; max.: maximum value. n: number of samples.

Table S3e. Main components of *L. stoechas* subsp. *luisieri* essential oil (Zone: 9. Serra de São Mamede. 10. Sierra de San Pedro).

	9				10			
	**μ ± σ**	**min.**	**max.**	**n**	**μ ± σ**	**min.**	**max.**	**n**
α-Pinene	1.46 ± 0.47	1.12	1.79	2	**1.80 ± 0.16**	1.56	1.95	5
Cymene Isomer	**2.87 ± 0.57**	2.46	3.27	2	**2.20 ± 1.16**	0.26	3.29	5
1.8-Cineole	0.62 ± 0.81	0.04	1.19	2	**2.03 ± 1.99**	0.49	5.35	5
Unknown I	0.79 ± 1.12	0.00	1.58	2	1.38 ± 0.78	0.00	1.95	5
Fenchone	0.63 ± 0.89	0.00	1.26	2	0.56 ± 0.46	0.00	1.04	5
Camphor	**3.49 ± 1.99**	2.08	4.90	2	1.86 ± 0.54	0.98	2.45	5
Linalool	**4.37 ± 0.54**	3.98	4.75	2	**4.59 ± 0.57**	3.99	5.25	5
trans-α-Necrodyl Acetate	**18.33 ± 1.06**	17.58	19.08	2	**24.25 ± 2.15**	21.22	26.83	5
Lavandulyl Acetate	**4.55 ± 1.17**	3.72	5.37	2	**4.62 ± 0.70**	4.02	5.80	5
cis-α-Necrodyl Acetate	1.39 ± 0.01	1.38	1.39	2	1.59 ± 0.28	1.17	1.92	5
Arbozol	**1.94 ± 0.89**	1.31	2.57	2	1.74 ± 0.48	1.10	2.43	5
5-Methylene-2,3,4,4-tetrame-2-Cyclopentenone	**2.96 ± 0.30**	2.74	3.17	2	**3.01 ± 0.39**	2.56	3.41	5
Trans-α-Necrodol	**7.19 ± 1.44**	6.17	8.21	2	**8.48 ± 2.84**	5.30	11.80	5
cis-α-Necrodol	1.62 ± 0.06	1.58	1.66	2	**1.86 ± 0.18**	1.63	2.00	5
Unknown X	0.76 ± 1.07	0.00	1.52	2	0.37 ± 0.84	0.00	1.87	5
Unknown XI	1.00 ± 1.41	0.00	2.00	2	1.27 ± 0.77	0.00	1.85	5
Eudesma-3,7(11)-diene	0.90 ± 0.33	0.66	1.13	2	1.12 ± 0.72	0.47	2.29	5
Unknown XIII	1.00 ± 1.41	0.00	2.00	2	1.64 ± 1.10	0.00	2.84	5
Unknown XIV	1.80 ± 2.55	0.00	3.60	2	0.32 ± 0.44	0.00	0.87	5
Unknown XVI	**2.24 ± 0.83**	1.65	2.83	2	1.23 ± 0.92	0.00	2.20	5
Viridiflorol	**3.10 ± 0.20**	2.96	3.24	2	**1.89 ± 0.21**	1.68	2.16	5
Unknown XX	0.46 ± 0.64	0.00	0.91	2	0.57 ± 0.34	0.00	0.83	5
Copaborneol	1.48 ± 0.14	1.38	1.58	2	0.90 ± 0.12	0.79	1.07	5
α-Cadinol					0.94 ± 1.34	0.02	3.27	5

Note. μ ± σ: plus or minus one standard deviation from the mean. min.: minimum value; max.: maximum value. n: number of samples.

Table S3f. Main components of *L. stoechas* subsp. *luisieri* essential oil (Zone: 11. Sierra de Aracena y Picos de Aroche. 12. Sierra Morena de Sevilla).

	**11**				**12**			
	**μ ± σ**	**min.**	**max.**	**n**	**μ ± σ**	**min.**	**max.**	**n**
α-Pinene	1.39 ± 0.52	0.84	2.12	9	1.42 ± 0.28	0.91	1.77	8
Cymene Isomer	**2.11 ± 0.64**	0.57	2.68	9	**2.33 ± 0.30**	1.80	2.76	8
1.8-Cineole	**7.77 ± 5.44**	0.46	17.71	9	**15.96 ± 7.59**	1.59	24.24	8
Unknown I	1.50 ± 0.58	0.00	2.06	9	1.28 ± 0.55	0.00	1.71	8
Fenchone	0.16 ± 0.19	0.00	0.45	9	0.70 ± 0.68	0.00	2.07	8
Camphor	1.04 ± 0.36	0.40	1.53	9	**1.70 ± 1.08**	0.66	3.31	8
Linalool	**3.33 ± 0.92**	2.29	4.99	9	**2.65 ± 0.79**	1.64	4.27	8
trans-α-Necrodyl Acetate	**23.24 ± 4.06**	17.54	30.50	9	**18.68 ± 3.56**	12.16	23.76	8
Lavandulyl Acetate	**5.06 ± 0.85**	4.01	6.53	9	**3.94 ± 0.54**	3.11	4.93	8
cis-α-Necrodyl Acetate	1.49 ± 0.20	1.16	1.76	9	1.40 ± 0.32	0.84	1.70	8
Arbozol	1.84 ± 0.33	1.33	2.24	9	**1.68 ± 0.39**	1.13	2.20	8
5-Methylene-2,3,4,4-tetrame-2-Cyclopentenone	**3.13 ± 0.56**	2.37	3.99	9	**3.01 ± 0.56**	2.31	3.76	8
Trans-α-Necrodol	**8.40 ± 1.79**	5.92	10.89	9	**7.79 ± 1.80**	5.14	10.93	8
cis-α-Necrodol	1.76 ± 0.21	1.34	1.98	9	**1.68 ± 0.23**	1.29	1.99	8
Unknown X	0.21 ± 0.62	0.00	1.87	9	0.23 ± 0.66	0.00	1.86	8
Unknown XI	1.44 ± 0.61	0.00	2.10	9	1.44 ± 0.67	0.00	2.20	8
Eudesma-3,7(11)-diene	0.85 ± 0.44	0.14	1.77	9	0.65 ± 0.43	0.12	1.39	8
Unknown XIII	**2.37 ± 0.98**	0.00	3.56	9	**1.79 ± 1.07**	0.00	3.71	8
Unknown XIV	0.13 ± 0.38	0.00	1.15	9	0.08 ± 0.23	0.00	0.66	8
Unknown XVI	**1.96 ± 0.55**	0.82	2.87	9	1.47 ± 0.73	0.47	2.89	8
Viridiflorol	**2.09 ± 0.41**	1.58	2.81	9	**1.96 ± 0.46**	1.25	2.72	8
Unknown XX	0.80 ± 0.36	0.00	1.33	9	0.62 ± 0.38	0.00	1.20	8
Copaborneol	0.89 ± 0.40	0.00	1.48	9	0.95 ± 0.23	0.57	1.38	8
α-Cadinol	0.01 ± 0.04	0.00	0.12	9	0.64 ± 1.80	0.00	5.10	8

Note. μ ± σ: plus or minus one standard deviation from the mean. min.: minimum value; max.: maximum value. n: number of samples.

Table S3f. Main components of *L. stoechas* subsp. *luisieri* essential oil (Zone: 13. Vegas Bajas. 14. Villuercas).

	13				14			
	**μ ± σ**	**min.**	**max.**	**n**	**μ ± σ**	**min.**	**max.**	**n**
α-Pinene	**2.47**			1	**2.83 ± 0.57**	2.42	3.23	2
Cymene Isomer	**2.68**			1	**2.39 ± 0.35**	2.14	2.64	2
1.8-Cineole	0.95			1	**11.09 ± 12.38**	2.33	19.84	2
Unknown I	1.72			1	1.31 ± 0.28	1.11	1.50	2
Fenchone	0.46			1	**5.18 ± 0.33**	4.95	5.41	2
Camphor	**2.02**			1	**2.84 ± 0.87**	2.22	3.45	2
Linalool	**4.25**			1	**2.12 ± 0.85**	1.52	2.72	2
trans-α-Necrodyl Acetate	**20.90**			1	**16.45 ± 2.88**	14.41	18.49	2
Lavandulyl Acetate	**4.28**			1	**3.85 ± 1.20**	3.00	4.69	2
cis-α-Necrodyl Acetate	1.72			1	1.12 ± 0.14	1.02	1.22	2
Arbozol	**2.43**			1	1.49 ± 0.10	1.42	1.56	2
5-Methylene-2,3,4,4-tetrame-2-Cyclopentenone	**3.52**			1	**2.52 ± 0.89**	1.89	3.15	2
Trans-α-Necrodol	**8.07**			1	**6.27 ± 2.76**	4.32	8.22	2
cis-α-Necrodol	1.93			1	1.65 ± 0.54	1.27	2.03	2
Unknown X	0.00			1				
Unknown XI	1.69			1	1.74 ± 0.54	1.36	2.12	2
Eudesma-3,7(11)-diene	0.98			1	1.20 ± 0.33	0.97	1.43	2
Unknown XIII	1.65			1	1.46 ± 0.21	1.31	1.61	2
Unknown XIV	0.00			1				
Unknown XVI	1.27			1	1.17 ± 0.16	1.05	1.28	2
Viridiflorol	2.01			1	1.68 ± 0.38	1.41	1.95	2
Unknown XX	0.39			1	0.48 ± 0.18	0.35	0.60	2
Copaborneol	0.93			1	0.82 ± 0.18	0.69	0.95	2
α-Cadinol	**3.46**			1	1.71 ± 1.15	0.90	2.52	2

Note. μ ± σ: plus or minus one standard deviation from the mean. min.: minimum value; max.: maximum value. n: number of samples.
